# Integrated Proteomic Analysis of Human Cancer Cells and Plasma from Tumor Bearing Mice for Ovarian Cancer Biomarker Discovery

**DOI:** 10.1371/journal.pone.0007916

**Published:** 2009-11-19

**Authors:** Sharon J. Pitteri, Lellean JeBailey, Vitor M. Faça, Jason D. Thorpe, Melissa A. Silva, Reneé C. Ireton, Marc B. Horton, Hong Wang, Liese C. Pruitt, Qing Zhang, Kuang H. Cheng, Nicole Urban, Samir M. Hanash, Daniela M. Dinulescu

**Affiliations:** 1 Fred Hutchinson Cancer Research Center, Seattle, Washington, United States of America; 2 Eugene Braunwald Research Center, Department of Pathology, Brigham and Women's Hospital, Harvard Medical School, Boston, Massachusetts, United States of America; University of Tuebingen, Germany

## Abstract

**Background:**

The complexity of the human plasma proteome represents a substantial challenge for biomarker discovery. Proteomic analysis of genetically engineered mouse models of cancer and isolated cancer cells and cell lines provide alternative methods for identification of potential cancer markers that would be detectable in human blood using sensitive assays. The goal of this work is to evaluate the utility of an integrative strategy using these two approaches for biomarker discovery.

**Methodology/Principal Findings:**

We investigated a strategy that combined quantitative plasma proteomics of an ovarian cancer mouse model with analysis of proteins secreted or shed by human ovarian cancer cells. Of 106 plasma proteins identified with increased levels in tumor bearing mice, 58 were also secreted or shed from ovarian cancer cells. The remainder consisted primarily of host-response proteins. Of 25 proteins identified in the study that were assayed, 8 mostly secreted proteins common to mouse plasma and human cancer cells were significantly upregulated in a set of plasmas from ovarian cancer patients. Five of the eight proteins were confirmed to be upregulated in a second independent set of ovarian cancer plasmas, including in early stage disease.

**Conclusions/Significance:**

Integrated proteomic analysis of cancer mouse models and human cancer cell populations provides an effective approach to identify potential circulating protein biomarkers.

## Introduction

Proteins detectable in serum and plasma are commonly relied upon to monitor ovarian, pancreatic, and colon cancer response to therapy and disease recurrence through the measurement of CA125, CA19.9, and CEA respectively. In addition, the screening and monitoring of prostate cancer currently relies in part on measurements of PSA levels in blood [1]–[3]. The development of effective strategies for identification of circulating protein markers that complement current markers would be beneficial [4]. A number of recent ovarian cancer studies have utilized proteomics to identify proteins in ovarian cancer cells, tissues, and fluids [5]–[9]. Such studies suggest hundreds of potential candidates on account of overexpression, but do not give an indication as to whether increased blood levels of the candidate proteins may occur in cancer subjects. The identification of novel circulating protein markers through plasma profiling represents a substantial challenge. Although plasma is one of the most accessible biological materials it contains vast assemblies of proteins and complexes and exhibits considerable heterogeneity between and within subjects that hinder proteomic analysis of low abundance proteins [10]. Engineered mouse models of cancer characterized by limited heterogeneity and a favorable tumor to body mass ratio, and isolated tumor cell populations that may be profiled at substantial depth present alternative strategies for the identification of potential cancer markers, notably secreted proteins that may be subjected to validation in human blood using sensitive assays.

We have developed several mouse models of epithelial ovarian cancer [11]. These models have been generated using intrabursal delivery of Adeno-Cre (AdCre) adenovirus via the infundibulum in genetically engineered mice. This method selectively activates oncogenes and inactivates tumor suppressors within the ovarian surface epithelium (OSE), a site of origin for many human ovarian tumors. We previously relied on the *LSL-K-ras^G12D/+^* and *Pten^loxP/loxP^* conditional murine strains (herein referred to as K-ras/Pten) to develop a mouse model of ovarian cancer [11]–[13]. A second model developed in parallel by us and others is based on the cooperation between the Wnt and Pten pathways (*APC^loxP/lox^ Pten^loxP/lox^* genetic combination, herein referred to as Pten/Apc) [14]. Both models exhibit features of endometrioid ovarian tumors.

We have recently undertaken proteomic profiling of ovarian cancer cell populations including cell lines and fresh tumor cells enriched from ascites fluid, which resulted in the identification of several thousand proteins and elucidated the repertoire of proteins expressed on the cell surface and proteins released into the extra-cellular milieu [15]. Proteome analysis has uncovered shedding of extra-cellular domains and highly dynamic processes of protein secretion. Here we have applied an in-depth quantitative proteomic approach [16]–[18] to the analysis of plasma protein changes related to tumor development in a K-ras/Pten ovarian cancer mouse model to determine their involvement in pathways and networks and their correspondence to proteins expressed or released from human ovarian cancer cells. Blinded analysis of human samples was done to determine which assayed proteins from the integrated cancer cell and mouse plasma data yielded statistically significant increases in their levels in ovarian cancer cases relative to controls. A protein subset representing primarily secreted proteins from the combined mouse plasma and human cancer cell proteomic data yielded significant differences in levels between plasmas from ovarian cancer patients and plasmas from control subjects.

## Results

### Quantitative Plasma Protein Changes Observed in an Ovarian Cancer Mouse Model

**Figure 1:**
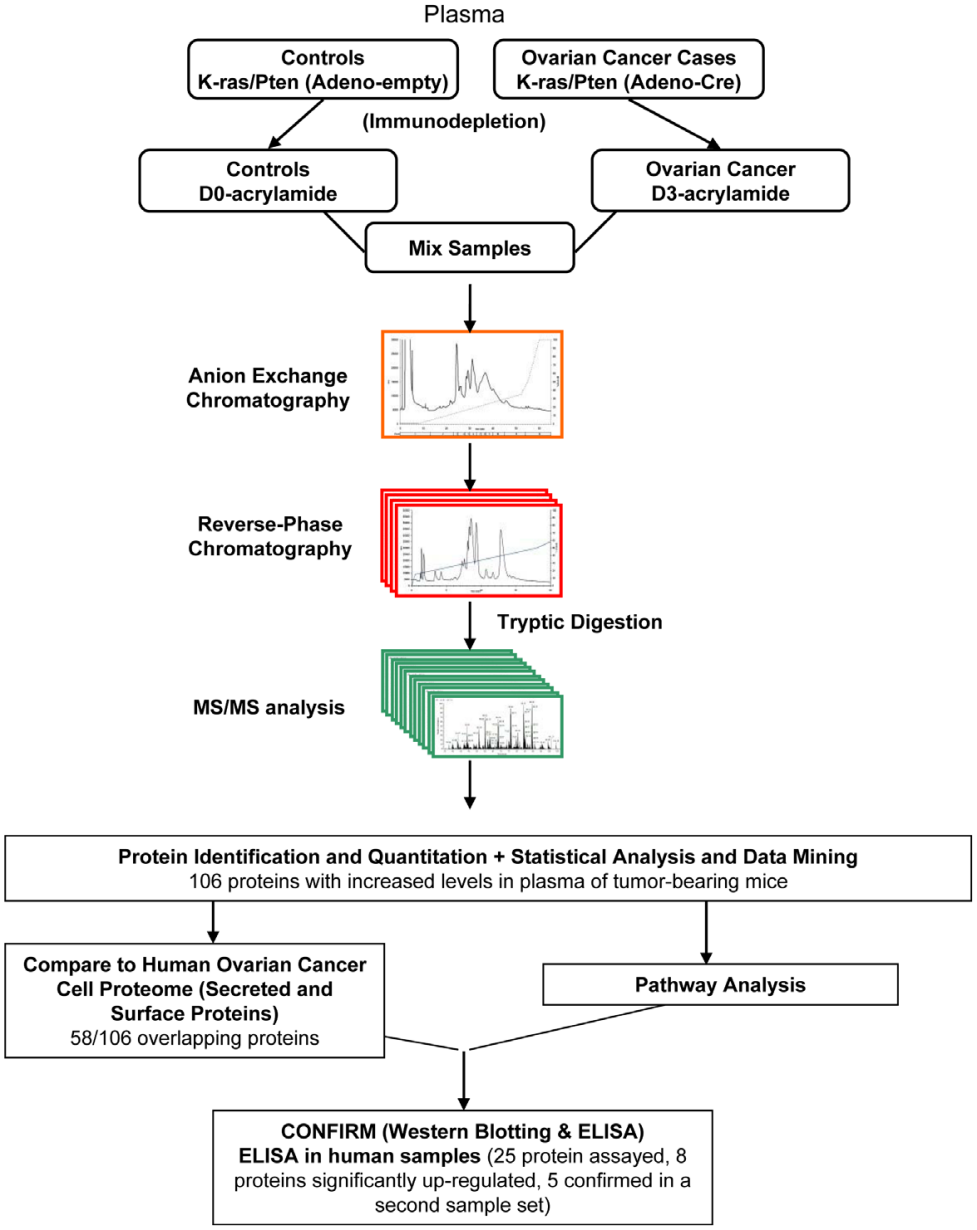
Study Design. A schematic of the workflow used in this study. Pools of control and cancer plasma from the K-ras/Pten mouse model were first immunodepleted to remove abundant proteins and then labeled with D0- and D3- acrylamide isotopes to distinguish cancer from control. The pools were mixed and subjected to extensive intact protein fractionation by anion exchange, followed by reverse phase chromatography. After tryptic digestion, samples were subjected to high resolution mass spectrometry and shotgun LC-MS/MS analysis for protein identification and quantitation. Following statistical analysis and data mining, upregulated proteins in murine plasma were compared to human ascites derived tumor cells/cancer cell line data. In addition, pathway analysis was used to determine significant biological pathways and processes. The expression of relevant upregulated proteins was further validated in mouse and human tumors and plasma.

A pool of plasma samples from AdenoCre-infected K-ras/Pten mice (n = 5) and a pool from Adeno-empty injected controls (n = 5) were subjected to quantitative proteomic analysis to determine differences in plasma protein levels. Separate pools of plasma from cases and controls were subjected to immunodepletion to remove abundant plasma proteins, followed by differential isotopic labeling to distinguish cancer cases from controls. The samples were mixed and then subjected to intact protein fractionation by ion exchange followed by reverse phase chromatography. Proteins in the individual collected fractions were enzymatically digested and subjected to online LC-MS/MS for protein identification and quantification (Figure 1). A feature of the IPAS platform is that extensive fractionation allows de-complexing of the samples into individual fractions to allow identification and quantification of proteins present in the plasma over 6–7 orders of magnitude [16], [19]. A previous study using this approach has shown that quantitative analysis of protein changes can be reliably determined [18]. In this study, some 1,725,000 mass spectra were collected and analyzed. 1,031 unique proteins were identified with high confidence (Table S1). 106 proteins were upregulated 1.5-fold or greater (p-value<0.05) (Table S2). A majority (57%) of the upregulated proteins contained a signal peptide for secretion, whereas 17% encompassed in their corresponding gene sequence a trans-membrane domain. 28% of the upregulated proteins were previously identified in proteome profiling of mouse liver tissue, a major source of plasma proteins [20], [21]. 9% had no human ortholog as determined based on the Mouse Genome Database [22]. In contrast, 36 proteins were downregulated 1.5-fold or greater (p-value<0.05) (Table S3) including secreted proteins with 8 representing proteins previously identified in mouse liver tissue.

### Comparative Analysis of Tumor Bearing Mouse Plasma and Human Ovarian Tumor Cell Proteomes

We compared the mouse plasma data with data from an extensive proteomic study of three human ovarian cancer cell lines (OVCAR3, CAOV3, and ES2) and of tumor cells from ascites fluid obtained from an ovarian cancer patient [15]. The cell lines consisted of two poorly differentiated serous adenocarcinomas (OVCAR3, CAOV3) and one clear cell carcinoma (ES2). The ascites derived tumor cells were collected from a patient with serous ovarian cancer. Cells were isotopically labeled in culture using SILAC [23] to allow ascertainment of the cellular origin of proteins identified in media. By comparing the upregulated mouse plasma proteins with the list of proteins enriched in the surface or secreted cellular compartments from human ovarian cancer cells (Table S2), 55% (58/106) of upregulated proteins in mouse plasma were found to be released from ovarian cancer cells through secretion or shedding or enriched in the ovarian cancer cell surface compartment. Previously described candidate markers for ovarian cancer identified in both the mouse plasma and ovarian cancer cells (Table S4), included WFDC2 (HE4), IGFBP2, and LCN2. The balance of proteins not identified in ovarian cancer cell analyses consisted primarily of inflammatory and immune response related proteins. Interestingly, although the K-ras/Pten model has features of endometrioid ovarian cancer, the data suggested that upregulated secreted proteins identified in mouse plasma with tumor development were more broadly representative of other ovarian cancer histological subtypes, since the human cell lines and ascites derived tumor cells resulted from papillary adenocarcinoma, clear cell and serous subtypes respectively [24], [25].

In addition to the quantitative cancer-to-control ratios for proteins presented, several proteins had labeled peptides only detected with the heavy form of acrylamide, representing “cancer-only” peptides. Of the 106 proteins up-regulated in mouse plasma IPAS, 33 proteins had additional cancer-only peptides identified (Table S2), further corroborating their up-regulated status. Additionally, seven proteins: Cacna1c, Vcl, Fcgbp, Mmp19, Lyz2, Eef1a1, and Gapdhs had cancer-only peptides (Table S2). Vcl acts as a linking protein in focal adhesion and has been studied within the context of cancer [26]. Mmp19, part of the metalloproteinase family implicated in cancer, has been reported to induce epithelial cell migration, playing an important role in the early stage of cancer [27]. Members of the Eef1a family have been reported as putative oncogenes overexpressed in ovarian cancer [28], [29].

### Biological Functions and Networks among Upregulated Proteins in Mouse Plasma

Pathway analysis was performed to determine the biological processes that contribute changes to the plasma proteome with ovarian tumor development, and explore pathways for the proteins of interest to understand the processes that they participate in. We relied on two pathway tools to this effect, Ingenuity and GeneGo's MetaCore. Initial pathway analysis of the list of 106 upregulated proteins using the computational gene network prediction tool, Ingenuity Pathways Analysis, categorized the proteins into biological functions. Thirty proteins were associated with inflammation (p = 1.30×10^−6^). Cancer was identified as a significant disease process (p = 3.71×10^−5^) in relation to all proteins identified, being represented by 52 relevant proteins from this list. Interestingly, genes for 11 proteins were associated as a group with ovarian cancer (p = 1.41×10^−3^) (CFH, CLU, IFI30, IGFBP2, IGFBP4, LCN2, MMP2, POSTN, TFF3, TNFRSF9, HE4 (WFDC2). HE4, a known candidate marker for ovarian cancer [30], [31] was upregulated (8.8-fold p<0.001) in the murine plasma data set. Some proteins from this list have also been identified in tumor studies as clinical outcome predictors (NOV, CLU TNC, POSTN, IGFBP2, LCN2) or mediators of resistance to chemotherapy (CLU, FBLN1, THBS, STIP1, PTGES3, IGFBP4, ATOX1) [32]–[45].

**Figure 2:**
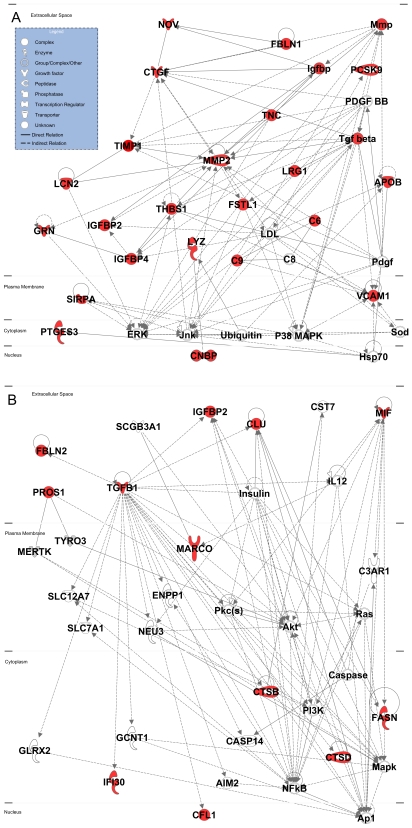
Networks. Top two networks (A, B) assigned by Ingenuity Pathway Analysis for upregulated proteins in the mouse plasma that were also enriched in the cancer cell line data. Central nodes include TGFβ, PI3K, MMP-2, Ras, and MAPK. Proteins colored in red represent upregulated proteins and non-colored proteins are those assigned by Ingenuity databases as possible intermediate interactions as based on the Ingenuity database. Solid lines indicate direct relationships (two molecules make physical contact) and dotted lines indicate indirect relationships (does not require physical contact). The scores for A) and B) are 51 and 24 respectively.

58 of the 106 upregulated proteins in the mouse plasma analysis were found to be also enriched in the surface or secreted sub-cellular compartments of ovarian cancer cells. Pathway analysis of these 58 proteins identified four significant networks using Ingenuity (Figure 2 and Figure S1) and five networks with MetaCore's workflow (Figure S2). The most prevalent processes highlighted in the Ingenuity networks included tumor growth, cell proliferation and apoptosis, regulation of tumor microenvironment, angiogenesis, cellular migration, invasion, and metastasis (Table S5). An additional assessment using MetaCore's enrichment tools across GeneGo ontologies and Gene Ontology (Figure S2, Figure S3, Table S6) confirmed cell adhesion, proliferation, development and extracellular matrix remodeling as significant represented processes. The top two networks generated by Ingenuity underscore the importance of TGFβ-mediated regulation of cell proliferation, metabolism and cytoskeletal remodeling (Figure 2, Table S5), complementing the MetaCore enrichment results (Figure S3). In addition to TGFβ, other central nodes (with both Ingenuity and MetaCore) include MMP2, p38MAPK, NFkB, and RAS, all known to be important in ovarian cancer [46], [47]. Pathway analysis of the 48 proteins that were found to be upregulated in mouse plasma with tumor development, but not identified as secreted or surface membrane proteins in ovarian cancer cells yielded three significant networks with Ingenuity (Figure S4, Table S5). The list included putative inflammatory proteins such as haptoglobin (HP), S100A8, and CCL8. The categorization of upregulated proteins in the plasma based on enrichment in ovarian cancer cells sub-fractions provided a means for assessing which upregulated proteins in plasma were more likely derived from cancer cells, and which proteins were more likely related to host-response.

### Immunoblot Analysis of Mouse and Human Specimens

**Figure 3:**
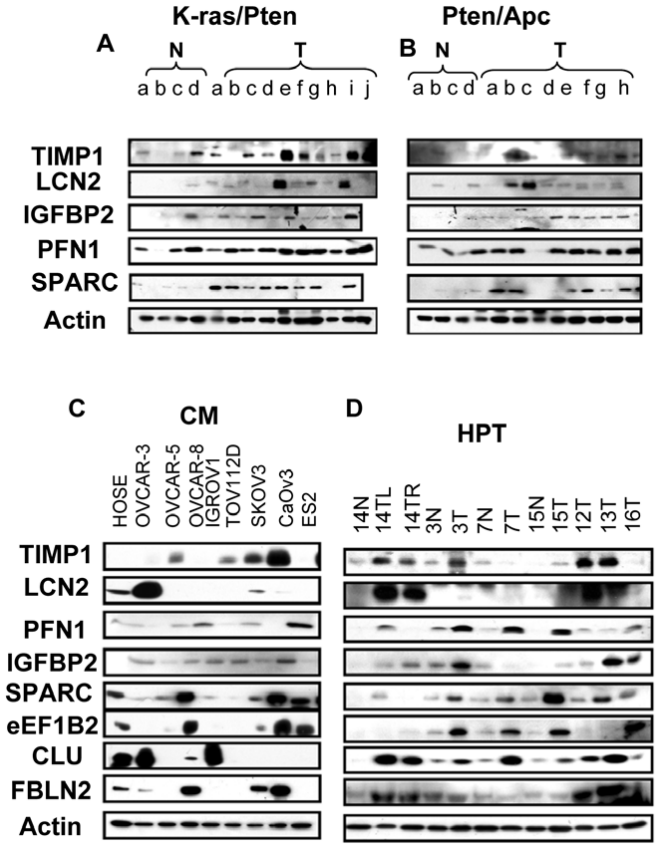
Validation of key tumor markers by western blotting. Protein levels of TIMP1, LCN2, PFN1, IGFBP2, SPARC, EEF1B2 and CLU were determined from the following: (A) normal ovarian tissue (N) and ovarian tumors (T) isolated from the K-ras/Pten, or (B) Pten/Apc mouse model of ovarian cancer, (C) conditioned media (CM) from normal human surface epithelium cells lines (HOSE) and ovarian cancer cell lines and (D) human primary tissues (HPT): normal ovarian tissue (N) and ovarian tumors (T). For mouse preparations, tissue lysates were prepared from normal ovaries and tumors taken from the same animal (a–d) along with four-five tumors from separate animals (e–i). For human primary tumor (HPT) samples, tissue lysates were prepared from 4 tumor samples paired with their respective normal tissue from the same patient and 3 lone tumor samples, in which no normal samples were available. Patient samples are as follows: patient #14 had independent bilateral serous borderline tumors in the left (TL) and right ovary (TR), tumors #3, #7, and #15 are papillary serous carcinomas, tumor #12 is an epithelial borderline tumor of the Müllerian-type, with endocervical mucinous and serous differentiation, tumor #13 is clear cell carcinoma, and tumor #16 is endometrioid adenocarcinoma.

A set of proteins identified in ovarian cancer cells and found to be upregulated in plasma from tumor bearing mice, TIMP1, LCN2, IGFBP2, PFN1, SPARC, EEF1B2, CLU, and FBLN2, were selected for immunoblot analysis using tumor tissue collected from mouse models, conditioned media from human ovarian cancer cell lines, and primary ovarian tumors from human subjects. Increased protein levels for Timp1, Lcn2, Igfbp2, Pfn1, and Sparc were observed in ovarian tumor tissues isolated from K-ras/Pten (Figure 3A) and Pten/Apc (Figure 3B) ovarian cancer mouse models compared to control tissue lysates. Similarly, enrichment in TIMP1, LCN2, IGFBP2, PFN1, SPARC, EEF1B2, CLU, and FBLN2 was observed in conditioned media (CM) collected from ovarian cancer cell lines derived from serous adenocarcinomas (OVCAR-3, SKOV3, CaOV3, OVCAR-5, OVCAR-8), endometrioid carcinoma (TOV112D), clear cell carcinoma (ES-2, IGROV1) compared to conditioned media derived from human ovarian surface epithelium (HOSE, Figure 3C). In addition, expression levels of TIMP1, LCN2, PFN1, IGFBP2, SPARC, EEF1B2, CLU, and FBLN2 were elevated in ovarian tumor lysates compared to control tissue freshly collected from patients undergoing surgery (Figure 3D).

### ELISA Assays Using Mouse and Human Plasmas

**Figure 4:**
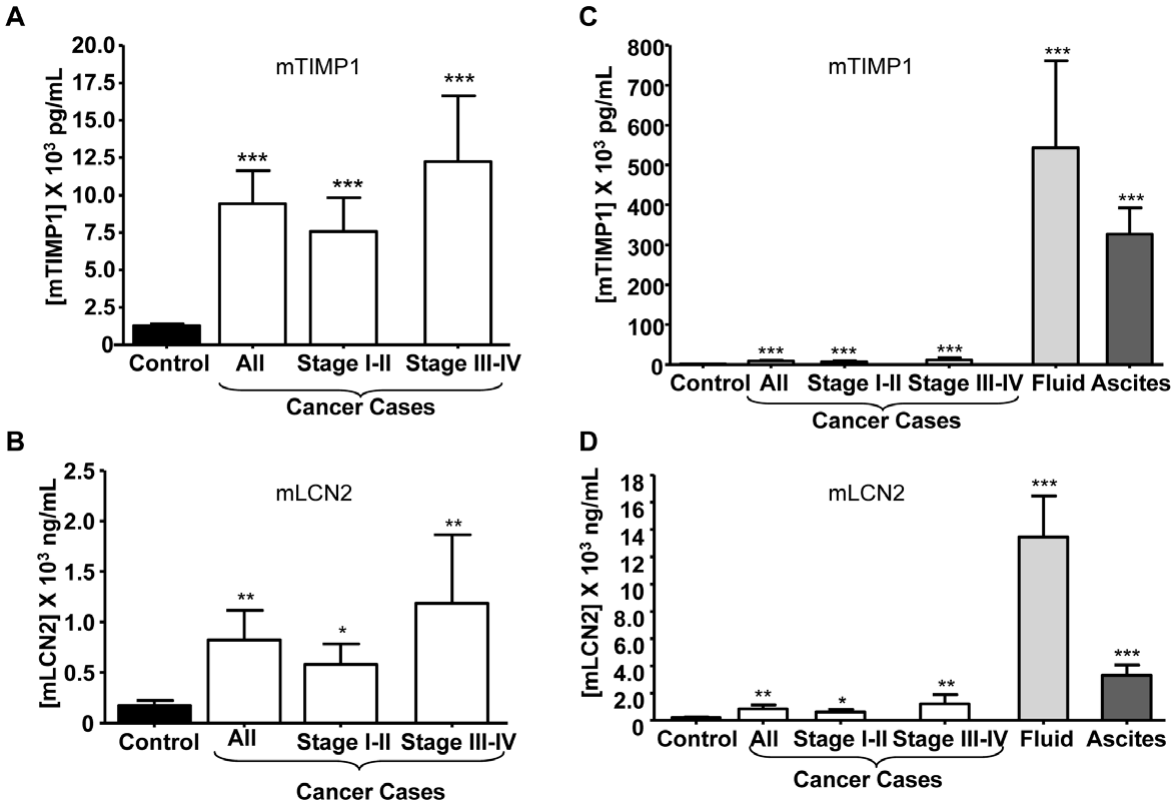
Plasma levels of mouse TIMP1 and LCN2 in cancer cases versus controls. TIMP1 and LCN2 levels in murine plasma at various stages of tumor progression were determined by ELISA as described in the Materials and Methods (A, B). Also shown are TIMP1 (C) and LCN2 (D) levels in murine ovarian tumor fluid and peritoneal ascites as compared to plasma. Statistical significance was determined using a two-tailed Student's t-test *p<0.05, **p<0.01, and *** p<0.0001 compared to controls.

We further tested a subset of proteins found by mass spectrometry to be upregulated in plasma from tumor bearing mice, for their levels in human and murine plasma. We also compared performance of proteins found to be secreted by ovarian cancer cells but were either not identified in mouse plasma or not found to be elevated in plasma from tumor bearing mice. 25 proteins were chosen based on availability of ELISA assays. For mouse assays, we assessed levels of Timp1 and Lcn2 in biological fluids and plasmas from tumor bearing mice (Stage I/II, n = 6; Stage III/IV, n = 5; controls, n = 18). Timp1 levels in Stage I/II and Stage III/IV samples were increased 5.9-fold (p<0.0001) and 9.5-fold (p<0.0001) compared to controls, respectively (Figure 4A). Similar findings were observed for Lcn2 (Figure 4B). We further examined whether Timp1 and Lcn2 were enriched in fluid extracted from late stage ovarian tumors and in peritoneal ascites given the proximity of these fluids to ovarian cancer cells and the secreted nature of the proteins. Timp1 levels were elevated more than 400-fold and 250-fold in ovarian tumor fluid and peritoneal ascites compared to murine plasma, respectively (Figure 4C). Likewise, Lcn2 had a similar pattern with a 79- and 19-fold increased levels in ovarian tumor fluid and peritoneal ascites, respectively (Figure 4D), consistent with their active secretion into surrounding fluids by tumor cells.

**Figure 5:**
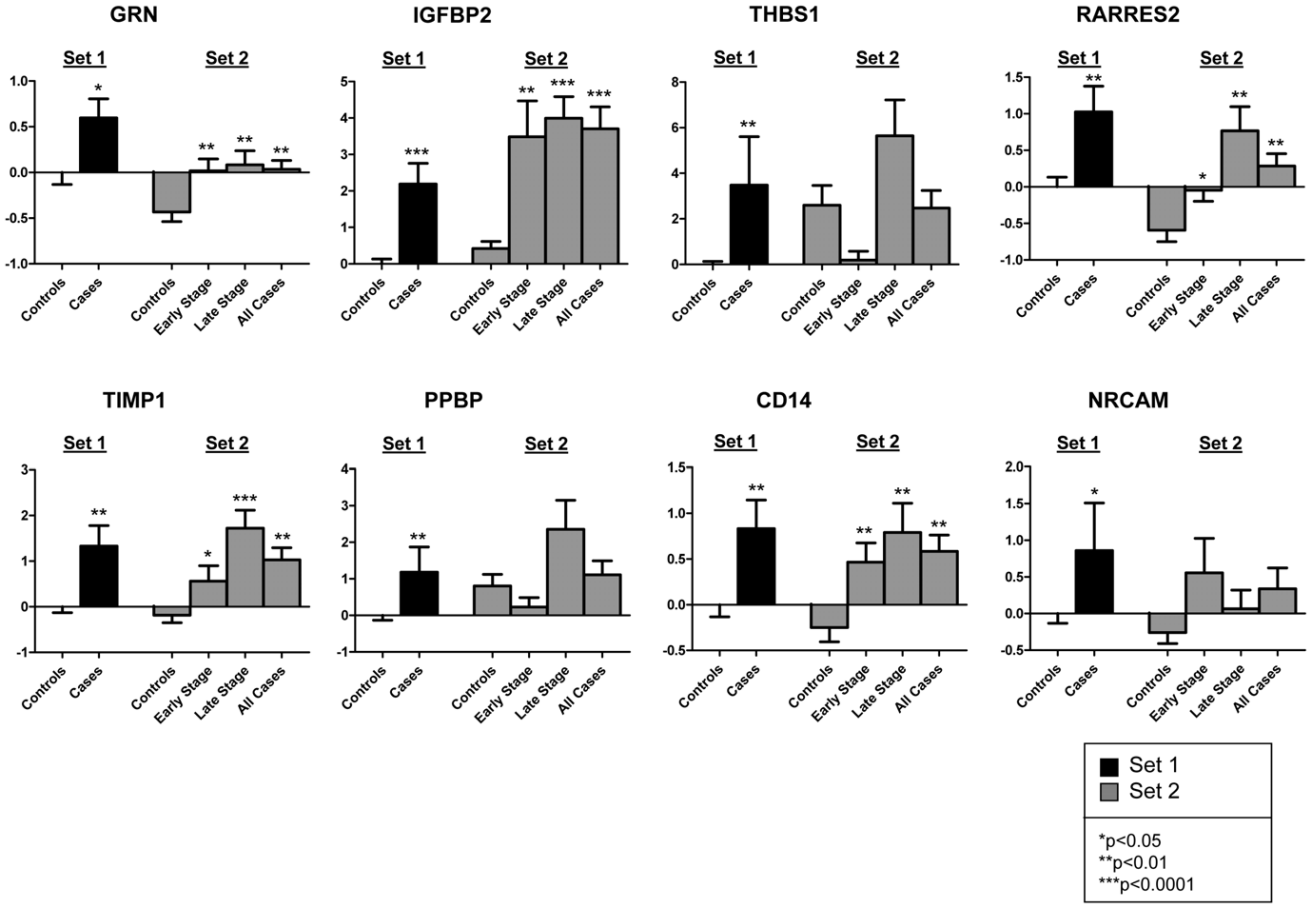
Plasma levels of human GRN, IGFBP2, THBS1, RARRES2, TIMP1, PPBP, CD14, and NRCAM in Set 1 and Set 2 samples. In Set 1, protein levels in cancer patients were compared to levels in the healthy controls, with all samples collected in the clinic under non-surgical conditions. In Set 2, protein levels in cancer patients were compared to controls with all samples collected in the operating room under surgical conditions. Logistic regression analysis was used to determine statistical significance of changes in protein levels between case and control groups. For Set 2, the p-values for the early and late stages were calculated if the p-value was significant in the entire Set 2 (all cases). *p<0.05, **p<0.01, and ***p<0.0001 for cases compared to controls. Marker levels were normalized to give healthy controls a mean of 0 and a standard deviation of 1. The y-axes represent the standardized marker levels.

The 25 proteins chosen for assays in human plasmas were either: 1) up-regulated in mouse plasma, 2) expressed by ovarian cancer cells, or 3) both up-regulated in mouse plasma and expressed by ovarian cancer cells. The initial set of human plasmas, henceforth referred to as Set 1, consisted of plasma samples from 13 women with epithelial ovarian cancer (n = 3 early stage, n = 10 late stage) and 56 healthy women. All samples in Set 1 were collected in the clinic, under non-surgical conditions (Table S7). Assay results from Set 1 are summarized in Table S2, together with the mouse plasma and cell line data used to choose the proteins for testing. The levels of 8 of the 25 proteins, GRN, IGFBP2, THBS1, RARRES2, TIMP1, PPBP, CD14, and NRCAM, were found to be statistically significantly (p<0.05) elevated in newly diagnosed subjects with ovarian cancer compared to controls (Table 1, Figure 5).

**Table 1:** Proteins increased in human ovarian cancer patient samples compared to control samples in Set 1* (p<0.05).

			ELISA SET 1* Logistic Regression										ELISA SET 2** Logistic Regression						
			Secreted/Total Extract				Surface/Total Extract				All Cases		All Cases		Early Stage Cases		Late Stage Cases		
Gene Name	Subcelluar Location	Mouse Plasma Cancer/Control Ratio	Ascites	OVCAR3	CAOV3	ES2	Ascites	OVCAR3	CAOV3	ES2	Coefficient	p-value	Coefficient	p-value	Coefficient	p-value	Coefficient	p-value	References to Prior Studies
**Mouse + Cell Line**																			
Grn	Extracellular	1.88	▪	↑	↑	↑	-	-	-	↓	0.595	**0.047**	0.465	**0.002**	0.449	**0.009**	0.487	**0.009**	-
Igfbp2	Extracellular	1.88	↑	↑	↑	↑	-	-	-	-	2.118	**0.000**	3.284	**0.000**	3.065	**0.001**	3.568	**0.000**	[39], [50]
Thbs1	Extracellular	2.12	↑	↑	↑	↑	↑	↑	-	↑	3.479	**0.001**	−0.126	0.915	-	-	-	-	[77]
Rarres2	Extracellular	3.07	↑	↑	↑	-	-	-	↑	-	1.027	**0.002**	0.877	**0.000**	0.546	**0.016**	1.304	**0.000**	-
Timp1	Extracellular	5.20	↑	↑	↑	↑	-	-	↑	↑	1.327	**0.000**	1.214	**0.001**	0.745	**0.040**	1.819	**0.000**	-
**Mouse Only**																			
Ppbp	Extracellular	1.67	-	-	-	-	-	-	-	-	1.181	**0.007**	0.303	0.568	-	-	-	-	-
**Cell Line Only**																			
CD14	Membrane	ID	↑	-	↑	-	↑	-	▪	↑	0.832	**0.010**	0.833	**0.001**	0.715	**0.007**	0.985	**0.003**	[51]
NRCAM	Membrane	-	↑	-	↑	-	-	↑	↑	-	0.860	**0.041**	0.598	0.102	-	-	-	-	-

* SET 1: Cases vs. Healthy Controls, *Non-Surgical Collections for All Cases and Controls*.

** SET 2: Cases vs. Controls n = 39, *Surgical Collections for All Cases and Controls*.

Bonferroni cutoff for multiple (25) comparisons p<0.002.

↑indicates increased >2-fold, ↓ indicates decreased >2-fold,

▪indicates <2-fold change.

For Set 2, the coefficients and p-values for the early and late stages are listed separately if the p-value was significant in the entire Set 2 (all cases).

Interestingly, 5 of the 8 proteins found up-regulated in Set 1 (GRN, IGFBP2, THBS1, RARRES2, and TIMP1) were secreted proteins from the intersection of mouse plasma and cancer cell data. PPBP was a secreted protein found up-regulated in the mouse plasma, but not discovered in the ovarian cancer cell lines, while CD14 and NRCAM were cell surface proteins from the ovarian cancer cell lines, and were not quantified in mouse plasma.

The 8 proteins that showed statistically significant increased levels in Set 1 assays were further tested in an additional set of human plasma samples, henceforth referred to as Set 2. Set 2 consisted of plasma samples from 55 women with epithelial ovarian cancer (n = 31 early stage, n = 23 late stage, and n = 1 stage unknown) and 39 controls undergoing surgery. All samples in Set 2 were collected in the operating room under surgical conditions (Table S7). Of the 8 proteins found significant in Set 1, 5 proteins (GRN, IGFBP2, RARRES2, TIMP1, and CD14) were confirmed to be up-regulated in Set 2 (Table 1, Figure 5). Notably all 5 of the proteins were statistically significantly elevated in the early stage samples.

Of the 25 proteins that were assayed, ten had concordant findings in mouse plasma and cell line data. Interestingly, five proteins that were present at the intersection of candidates from mouse plasma and cell population data (GRN, IGFBP2, THBS1, RARRES2, and TIMP1), were found to be significantly upregulated in Set 1 and four of the five proteins were confirmed in Set 2, with THBS1 as the exception.

Specimen collection conditions can affect the levels of circulating proteins. For instance, patients who have blood drawn at the time of surgery may have increased levels of certain proteins as a result of stress [48]. Using a previously described method [48], we evaluated whether protein levels differed between case and control groups after adjusting for conditions of blood collection. In the samples used for assays, seven ovarian cancer patients had blood draws at two time points: prior to surgery, and at the time of surgery. The protein levels at both time points for each patient are shown in Figure S5. Regression analyses were performed using Generalized Estimating Equations (GEE) methods [48] to account for the correlation in the protein levels of the 7 women with two blood collections, and give p-values that are unbiased by the multiple blood draws from the same women (Table S8). After adjusting for conditions of blood draw, the five proteins previously found significant in Set 1 and Set 2 logistic regression analysis, were also found significant in the GEE model for the case versus healthy control comparison. For a secondary GEE model, the coefficient for blood draw conditions were fixed from the first GEE analysis to avoid bias from refitting. The second analysis was restricted to specimens collected at surgery and all 5 proteins found significant in the first analysis were significant in the case vs. non-case, early case vs. non-case, and late case vs. non-case comparison. These results support the findings from the logistic regression and show that IGFBP2, TIMP1, RARRES2, CD14, and GRN are elevated in all cases compared to control and importantly in early cases compared to controls.

GRN has been described as a putative novel growth factor for ovarian cancer, and was found to be highly secreted by ovarian cancer cells [49]. Increased levels of GRN and RARRES2 in plasma from ovarian cancer patients in both the early and the late stage cases are a novel findings in this study. CD14, and IGFBP2 have been previously assayed in serum from ovarian cancer patients [37], [50], [51]. They were significantly elevated in this study in mouse plasma and enriched in the secreted protein fraction of ovarian cancer cell lines. A finding of interest in our study is the occurrence of increased levels of IGFBP2 and CD14 in early stage disease. TIMP1 performed also showed increased levels in both early and late stage cases. Cell line data indicated that TIMP1 was released from ovarian cancer cells at nanograms per million cancer cells per hour [18] which would account for increased levels in human ovarian cancer patient samples.

## Discussion

There is currently a limited understanding of the changes in plasma proteins that occur with the development of ovarian tumors and for most tumor types in general [52], [53]. The discovery of novel plasma markers has represented a substantial challenge, particularly for markers that are applicable to early stage disease. Analysis of high-dimensional genomic, transcriptomic or proteomic data allows for affected pathways, networks and signaling nodes to be explored [53], [54]. The present study provides evidence for the utility of integrating data from in-depth quantitative proteome analysis of mouse models of cancer with data from human cancer cells for biomarker identification. Here we used a mouse model of epithelial ovarian cancer in combination with the IPAS proteomic platform to allow us to reliably assess and quantify changes encompassing low abundance proteins in mouse plasma with tumor development [53]. Integration with data from human ovarian cancer cell lines provided a means for assessing which upregulated proteins were expressed in cancer cells. Additionally signaling nodes that contributed upregulated proteins in plasma were determined. As a result, proteins that likely resulted from inflammatory and immune response changes were distinguished from proteins that more likely resulted from secretion by tumor cells or from tumorigenic processes. Changes in tumor microenvironment and ECM are associated with autocrine regulation. ECM proteins have been previously identified as ovarian cancer metastasis signature genes [55], [56]. Changes in the cytoskeleton were observed including cytoskeletal-mediated migration, adhesion, and invasion. Lastly, cellular proliferation changes were observed and incorporated cell apoptosis. Several central signaling nodes were identified in this study including TGFβ, MMP2 and NFkB signaling. TGFβ, signaling in particular is central to a multitude of processes, including cell proliferation and apoptosis, ECM remodeling, cell migration, adhesion, invasion and metastasis, angiogenesis, and inflammation and immune surveillance [57], [58]. The TGFβ family is an active target for cancer prevention and therapy [57], [58]. Interestingly, TGFβ is known to have both tumor suppressor and pro-oncongenic effects in various cancers including ovarian [59]. TGFβ also plays a role in epithelial stem cell niche homeostasis [60], and deletion of TGFβ receptor induces a highly proliferative and invasive environment [61]. Tumbar et al. also determined that the loss of TGFβ receptors in combination with oncogenic Ras enhanced tumorigenicity. This hypothesis is also supported by the generated networks in our pathway analysis (Figure 2B), in which NFκB (presumably under the control of TGFβ) is assigned to elicit Ras, PI3K, and MAPK signaling towards actin-mediated responses driving cellular migration, EMT, invasion, and metastasis [59]. This role of TGFβ signaling is reminiscent of its effects on embryonic stem cell pluripotency and embryonic tissue development, including the ovary [62]–[64]. Consistent with this hypothesis, additional networks illustrated by pathway analysis (Figure S1) for the late stage proteome highlight Notch and Dkk3, known stem cell effectors [65], [66]. It is therefore not surprising that this effector is dominant in the plasma and cancer cell line data, supporting the current role of TGFβ as it elicits a variety of responses relevant to cancer processes (proliferation, apotosis, inflammation, angiogenesis, autorcrine-regulation of tumor microenvironment/ECM and adhesion, invasion, and metastasis).

In a search for potential ovarian cancer biomarkers, we performed a novel integrative analysis by comparing mouse plasma proteome data with human cell line and ascites derived tumor cell data. By using a mouse model to identify proteins of interest, extraneous sources of heterogeneity unrelated to disease were minimized. A large number of proteins common to plasma from tumor bearing cell lines and human ovarian cancer cells were found to be involved in cellular/tissue remodeling and cell-cell contact/communication that dictate alterations in ECM processes and facilitate cell migration, local tumor growth, and tumor metastasis. In this integrated analysis the subset of proteins that were most successfully validated in human ovarian cancer sera represented proteins found to be upregulated in plasma from tumor bearing mice and found to be secreted in human ovarian cancer cells. A lower percentage of proteins found to be upregulated in the mouse model or were found only in ovarian cancer cell data, yielded validated candidate markers in our set of human plasmas.

We chose the K-ras+/Pten(-) ovarian cancer model initially because of its histopathological representation of a subtype of ovarian cancer. However the finding of elevated levels in mouse plasma of proteins previously associated more broadly with ovarian cancer, led us to investigate the merits of integrating mouse plasma proteomic findings from this model with proteomic findings from ovarian cancer cells. The candidate proteins chosen for assays were tested in samples from human patients with a variety of ovarian cancer histologies. It is likely that these potential markers are broadly applicable to ovarian cancer and not confined to a specific histology or genetic subtype.

In this study, five proteins that exhibited significant differences in plasmas from subjects with ovarian cancer relative to controls were comprised predominantly of secreted proteins at the intersection of upregulated proteins in mouse plasma and secreted proteins in proteomic data from ovarian cancer cell populations. Further validation studies with these proteins as well as with additional candidates for which specialized assays are currently not available would be warranted. Integration of mouse model and cell line data as implemented in this study provides an innovative strategy that would be applicable to other cancer types for the discovery of circulating biomarkers.

## Materials and Methods

### Reagents and Antibodies

Anti-human and anti-mouse rat polyclonal LCN2 (1∶500), mouse monoclonal anti-human Clu (1∶500), and goat polyclonal anti-mouse Timp1 (1∶200) were purchased from R&D Systems (Minneapolis, MN US). Rabbit polyclonal anti-mouse/human EEF1B2 was purchased from Protein Tech (Chicago, Il US) rabbit polyclonal anti-mouse/human PFN (1∶2000) was purchased from Cell Signaling (Danvers, MA) and anti-mouse/human IGFBP2 (1∶500) was purchased from Upstate Biotechnology (Lake Placid, NY). Anti-human FBLN2 (1∶200), anti-human/mouse SPARC (1∶250), and anti-mouse/human TIMP1 (1∶250) were purchased from Santa Cruz Biotechnology (Santa Cruz, CA US). Anti-rat-horseradish peroxidase (HRP) conjugated secondary antibody (1∶2000) was purchased from Pierce Biotechnology Inc (Rockford, IL) and anti-mouse/rabbit/goat-HRP conjugated secondary antibodies were purchased from Vector Laboratories Inc (Burlingame, CA).

### Cell Culture

Human ovarian cancer cell lines OVCAR3, OVCAR5, OVCAR8, IGROV1, SKOV3 and TOV112D were maintained as 70% confluent monolayers in RPMI containing 10% FBS and 1% penicillin/streptomycin cocktail. Normal human ovarian surface epithelial lines (HOSE) and CaOV3 and ES-2 cancer cell lines were maintained in DMEM containing 10% FBS and 1% penicillin/streptomycin cocktail.

### Collection of Mouse Plasma

All animal studies were done in accordance with institutional guidelines and approved by the Harvard Medical School Animal Care and Use Committee. Generation of *LSL-K-ras^G12D/+^ Pten^loxP/loxP^* mice and adenoviral induction of ovarian tumors was accomplished as previously described [11]. Control mice were littermates that underwent the same surgical procedure but were injected with Adeno-empty virus instead of Adeno-Cre. Plasma was collected at a 10 week time point following Adeno-Cre (cancer cases n = 5) or Adeno-empty injection (controls n = 5). Cancer cases (10 weeks post-injection) had large ovarian tumors that had metastasized to pelvic or peritoneal locations. To collect plasma, mice were euthanized and blood was collected by cardiac puncture using a 22-gauge needle and 1 ml syringe. The blood was placed into a K3EDTA coated 1.5 ml microcentrifuge tube using a needless-syringe. After cardiac puncture, mice were surgically and pathologically examined to confirm the presence of ovarian tumors and metastases. Tumor volumes were calculated using the following formula (length×width×height)/2 according to published literature [67].

The collected murine blood was processed as follows: K3EDTA tubes containing the blood were centrifuged at room temperature for 10 minutes at 1500 rpm. The top layer of plasma was extracted from the separated blood mixture using a pipet, aliquoted, and frozen at −80°C. Plasma from 5 mice with cancer and 5 control mice were pooled for further analysis. 200 µl from each individual mouse was used to generate a pooled sample volume of 1 ml.

### Plasma Sample Depletion and Isotopic Labeling

Separate pools of cancer cases and controls were immunodepleted of albumin, IgG, and transferrin using a Ms-3 column (4.6×250 mm, Aglient, Wilmington, DE). The column was equilibrated with buffer A for 13 min at a rate of 0.5 ml/min. Pooled sera were filtrated through a 0.22 µm syringe filter and injected 75 µl at a time. Flow through fractions were collected for 10 minutes at a rate of 0.5 ml/min Buffer A. These fractions were combined and stored at −80°C until use. Material bound to the column was recovered by elution with buffer B for 8 min at a rate of 1 ml/min. Centricon YM-3 columns (Millipore) were used to concentrate the immunodepleted samples. These samples were then re-diluted in 8 M urea, 0.5 octyl-beta-d-glucopyranoside (Roche) and 30 mM Tris pH 8.5. Before labeling, each pooled sample was reduced with dithiothreitol (DTT) in 50 µl 2M Tris-HCl, pH 8.5 (0.66 mg DDT/mg protein). Next, isotopic labeling of intact proteins was accomplished by labeling the cysteine residues with acrylamide [68]. Control samples received the light acrylamide isotope (D0 acrylamide, Fluka), while cancer samples were labeled with the heavy 2,3,3′-D3-acrylamide isotope (D3 acrylamide, Cambridge Isotope Laboratories). Labeling was performed for 1 hour at room temperature. Pooled samples were either labeled with 7.1 mg of light acrylamide or 7.4 mg of heavy acrylamide per milligram of protein that had been diluted in a small volume of 2M Tris-HCl, pH 8.5.

### Protein Fractionation and Analysis by Mass Spectrometry

Following isotopic labeling, the cancer cases and control pools were mixed. The sample was fractionated identically in two dimensions: first by anion exchange (AEX) and second by reverse phase (RP). Fractionation was carried out as described previously [19], with a few modifications. Briefly, the mixed, labeled samples were diluted to 10 ml with 20 mM Tris in 6% isopropanol, 4 M Urea, pH 8.5, and injected immediately into a Mono-Q 10/100 column (Amersham Biosciences). The buffer system for the first dimensional fractionation scheme was solvent A: 20 mM Tris in 6% isopropanol, 4 M Urea pH 8.5 and solvent B: 20 mM Tris in 6% isopropanol, 4 M Urea, 1 M NaCl pH 8.5. The separation gradient was performed at a 4.0 ml/min flow rate as follows: 0 to 35% gradient of solvent B for 44 min, 35 to 50% gradient of solvent B for 3 min, 50 to 100% solvent B in 5 min, and a hold in solvent B at 100% for another 5 min. 65 fractions were collected and further pooled into 9 fractions (fractions 1–13, 14–24, 25–27, 28–30, 31–36, 37–39, 40–42, 43–45, and 46–65). These 9 pooled fractions were then separated by reverse phase fractionation on a Poros R2 column (4.6×50 mm, Applied Biosystems). Samples were fractionated in a TFA/acetonitrile buffer system as follows: Solvent A, composed of 95% H_2_0, 5% acetonitrile, and 0.1% TFA and Solvent B, composed of 90% acetonitrile, 10% H20, and 0.1% TFA. The flow rate was set at 2.7 ml/min and the following gradient was used: a desalting step of 5% Solvent B until the absorbance reached base line, 5 to 50% gradient of solvent B for 18 min, 50 to 80% solvent B for 7 min, and 80 to 95% solvent B in 2 min. Sixty-three 1.2 ml fractions were collected, thus a total of 567 fractions were generated from the entire two-dimensional fractionation. RP fractions from each of the 9 AEX fractions were pooled into 16 fractions (RP fractions).

In solution digestion with trypsin was performed on lyophilized aliquots of a total 144 pools. The samples were then subjected to shotgun LC-MS/MS analysis on a LTQ-FT (ThermoFisher Scientific) mass spectrometer equipped with a nano-LC system (Waters). The nano-LC was equipped with a 25 cm column (Picofrit 75 um ID, New Objective), packed in house with Magic C18 packing material (Michrom)). A 90-minute linear gradient was then applied from 5 to 40% acetonitrile in 0.1% formic acid at 300 nL/min. Spectra were acquired in data dependent mode with a MS1 *m/z* range of 400 to 1800, followed by selection of the 5 most abundant doubly or triply protonated ions in each MS1 spectrum for MS/MS analysis. The mass spectrometer parameters were as follows: capillary voltage of 2.1 kV, capillary temperature 200 degrees C, 100,000 resolution, and FT target value of 2,000,000.

### Protein Identification and Quantification

Data analysis was performed using the Computational Proteomics Analysis System [69]. Searches were performed using cysteine alkylation modification with the light form of acrylamide as a fixed modification and the heavy form of acrylamide (+3.01884) as a variable modification. Spectra were searched using X!Tandem [70] configured with the comet score module plug-in [71] against the mouse IPI database [72] version 3.29. A search for tryptic peptides was performed with a semitryptic refinement option where a second round of searching is performed for semitryptic peptides from proteins identified in the first round of searching.

Quantitative ratios were obtained for peptides containing cysteine residues labeled with heavy and light acrylamide isotopes. Quantitative information was extracted from acrylamide labeled peptides using an in-house script (Q3); this allowed us to obtain the relative quantification from MS1 spectra for each pair of peptides identified by MS/MS that contains cysteine residues [68]. Calculation of ratios between cancer and normal were fraction-centric (per LC-MS/MS run). All identified peptide measured acrylamide ratios were processed such that multiple measurements for a given peptide in one individual fraction were log_2_ averaged, resulting in a dataset containing one ratio per peptide per each individual fraction. A global normalization factor was then computed as the mode of the peptide ratio histogram. All peptide ratios for a specific protein present in a particular fraction were then normalized and log-averaged to obtain the local relative protein ratio. Statistical significance of protein quantitation was assigned by two methods as described below.

### Proteomic Data Analysis

Data was interrogated using Ingenuity Pathways Analysis (Ingenuity Systems®, www.ingenuity.com) and MetaCore from GeneGo Inc (www.genego.com). A dataset containing IPI accession numbers and the corresponding cancer-to-control ratios was uploaded into each application where all 1031 proteins identified in the IPAS experiment were used as a reference set. Each accession number was mapped to its corresponding gene object in the Ingenuity's knowledge base or MetaCore's manually curated data base. A fold change cutoff of 1.5 with a p-value<0.05, was set to identify genes whose expression was significantly differentially regulated. For analysis with Ingenuity, these genes, were designated as focus genes and were overlaid onto a global molecular network developed from information contained in the Ingenuity knowledge base. Networks of these focus genes were then algorithmically generated based on their connectivity. A score is generated for each network based on the fit between the focus genes and each network. The score is the –log(p-value) calculated based on a hypergeometric distribution with the right-tailed Fisher's Exact Test. For analysis with MetaCore, the gene list of proteins found to be up-regulated in the mouse plasma and secreted/shed in mouse and human cancer cell lines (total of 58 genes) was submitted to an enrichment and network workflow. Enrichment analysis was conducted across three GeneGo curated ontologies along with Gene Ontology [73] to provide a quantitative analysis of the most relevant biological functions represented by the data. Networks and the statistics for each, were generated using the analyze network algorithm, one of the nine network building algorithms in MetaCore.

PeptideProphet [74], an empirical statistical modeling program, was used to estimate the accuracy of peptide identifications. Factors determined by the search algorithm were weighted to assign a single number for each peptide identification that can be then compared to other peptide identifications. ProteinProphet [75] a program that applies a statistical model to infer protein groups from peptide identifications and validates these groups with a probability assignment, was also utilized. A protein group may contain one or more protein sequence, with each sequence being indistinguishable based on the identified peptides. Proteins with a ProteinProphet score corresponding to 5% error rate (∼3.5% false discovery rate as determined by ProteinProphet) were retained. In this study, for each protein group, henceforth referred to as “protein”, a representative gene symbol was chosen.

### Immunoblot Analysis

The expression pattern of key proteins from the IPAS analysis was analyzed in conditioned media (CM) of human ovarian cancer cell lines and human primary ovarian tumors (HPT) freshly collected from patients undergoing surgery. Human sample collection was approved by the Partners HealthCare Human Research Committee (Institutional Review Board), Harvard Medical School. In addition, western blot analysis was performed on ovarian tumors collected from two mouse models of ovarian cancer: K-ras/Pten and Pten/Apc.

Tissue homogenates were made with RIPA buffer (50 mM Tris-HCL, 150 mM NaCl, 1% NP-40, 0.5 C_24_H_39_NaO_4_, 0.1% SDS, pH 7.4) containing freshly added protease inhibitor cocktail Set I and II (Calbiochem, US) and Complete Mini Inhibitors (Roche, Indianapolis, US). Conditioned media (CM) was first concentrated for 30 min at 3000 rpm using Amicon Ulita-5 centrifugal filter device (Millipore, Billerica US) as described by the manufacturer. Samples were then sonicated or passed through a 27-gauge needle and protein concentrations were determined using a Bio-Rad system (Hercules CA, US). 10–20 µg of mouse primary tissue, 20 µg of HPT, or 40 µg human CM samples were prepared with RIPA buffer and 1X Laemmli sample buffer and then heated for 5 min at 100°C. Samples were resolved on 8, 12 or 15% SDS-PAGE gels and transferred to polyvinylidene difluoride membrane for 2 h. Membranes were subsequently blocked with 5% Milk in Tris-buffered saline containing 0.1% Tween-20 (20 mM Trisbase, 137 mM NaCl, pH 7.6) and incubated overnight at 4 deg C with primary antibodies, as specified in the text (for dilutions see Reagents and Antibodies). Horseradish peroxidase-conjugated secondary antibodies (1∶2000) were applied for 1 hour at room temperature and detection by chemiluminescence was performed using SuperSignal West Pico Chemiluminescent Substrate as specified by the manufacturer (Pierce Biotechnology Inc, Rockford, IL). Equal protein loading was assessed by probing for total actin protein.

### Enzyme-Linked Immunoabsorbant Assays

For validation studies using ELISA, we collected plasma from infected mice at various stages of tumor progression and controls. In addition, we also collected ascites or ovarian tumor fluid extracted from late stage tumors. Timp1 concentrations in murine plasma were measured using a Quanitikine-Mouse Timp1 ELISA Kit, while mouse Lcn2 levels were detected with a DuoSet ELISA Kit (R&D Systems, Minneapolis, MN USA). To measure Timp1, we diluted mouse plasma samples 1∶6, while a 1∶400 dilution was used for Lcn2. For data analysis, mice were grouped according to disease stage: control, Stages I-II (early stage) and Stage III-IV (late stage).

Human plasma samples were collected from women who consented to participate in a specimen donation protocol conducted by the Pacific Ovarian Cancer Research Consortium, which was approved by the Fred Hutchinson Caner Research Center Institutional Review Board. Each patient provided written informed consent. 163 samples were obtained in total consisting of: 68 women newly diagnosed with ovarian cancer (55 collected at the time of surgery, 13 collected in advance of surgery), 56 healthy controls (collected in the clinic from apparently healthy women attending regular breast cancer screening exams), and 39 surgical controls (11 patients undergoing gynecologic surgery for a variety of conditions but with normal ovarian pathology, and 28 samples from patients with benign ovarian disease collected at the time of surgery). The same specimen processing protocol was used for all samples. Human plasma levels of ADAM17 (1∶4), TNFRSF21 (1∶20), PI3 (1∶10), LGMN (1∶25), AXL (1∶250), IGFBP2 (1∶250), RARRES2 (1∶250), DKK3 (1∶300), ALCAM (1∶350), HGFR (1∶1000), CD14 (1∶2000), XLKD1 (1∶2000), VCAM1 (2500), NrCAM (1∶20), CDH1 (50), PPBP (1∶1000), IGF1R (1∶2) and NOV (1∶20) were evaluated using DuoSets, while plasma concentrations of TIMP1 (1∶100), THBS1 (1∶100) and TGFβ1 (1∶40) were measured using Quantikine kits (all purchased from R&D Systems, Minneapolis, MN, USA). Plasma levels of vWF (1∶100) (American Diagnostics, Stamford, CT, USA), GRN (1∶200) (Adipogen, Seoul, South Korea), sICAM2 (1∶20) (Abcam, Cambridge, MA, USA), and LCN2 (1∶500) (BioPorto Diagnostics, Gentofte, Denmark) were also measured. All assays using human plasma were done with sample clinical characteristics blinded.

### Statistical Analysis

Statistical significance of protein quantification by mass spectrometry was determined by two methods. Proteins for which multiple paired MS events of heavy and light acrylamide were observed, a one-sample t-test was used to calculate a p-value for the mean ratio of the whole protein across all fractions. Secondly, the probability for the ratio for each MS event was calculated from the distribution of ratios in a control-control experiment in which the same sample was labeled with heavy and light acrylamide. If the p-value for each individual event was <0.05, the overall protein ratio was considered statistically significant.

For ELISA measurements, protein levels were normalized to eliminate batch-to-batch variation in measurements. Marker levels were normalized to give healthy controls a mean of 0 and a standard deviation of 1 [76]. Different groups were compared using logistic regression analysis. In addition, regressions were performed to evaluate which proteins differed between case and control groups after adjusting for blood collection conditions using Generalized Estimating Equations methods. The Bonferroni adjustment was used to account for multiple testing using a value of 25 for the number of comparisons (since 25 proteins were assayed).

Data for the mouse plasma experiments has been deposited in the Mouse Peptide Atlas (www.peptideatlas.org/repository). Sample accession number PAe000322 [77].

## Supporting Information

Figure S1 Additional networks for upregulated in mouse plasma and enriched in ovarian cancer cell data. The remaining significant networks for proteins upregulated in mouse plasma and enriched in cancer cell data assigned by Ingenuity Pathway Analysis are shown. Proteins colored in red represent proteins from the IPAS list. Non-colored proteins are those assigned by the Ingenuity database as possible intermediate interactions. Solid lines indicate direct relationships (two molecules make physical contact) and dotted lines indicate indirect relationships (does not require physical contact). The scores for A) and B) are 51 and 24 respectively.
(0.38 MB PDF)

Figure S2 Significant networks for upregulated in mouse plasma and enriched in ovarian cancer cell data (by Metacore analysis). Five significant networks for proteins upregulated in mouse plasma and enriched in cancer cell data assigned by Metacore analysis are shown. Proteins with a pink dot represent proteins from the IPAS list. The p-values for each network are: A) 5.73e-24, B) 5.73e-24, C) 5.73e-24, D) 5.73e-24, E) 5.02e-15.
(0.75 MB PDF)

Figure S3 A) Gene Ontology and B) GeneGO processes for the 58 proteins upregulated in mouse plasma and enriched in ovarian cancer cell data.
(0.13 MB PDF)

Figure S4 Significant networks for proteins upregulated in mouse plasma and not found enriched in ovarian cancer cell data. The significant networks for proteins upregulated in mouse plasma, but not enriched in cancer cell data assigned by Inenuity Pathway Analysis are shown. Proteins colored in red represent proteins from the IPAS list. Non-colored proteins are those assigned by the Ingenuity database as possible intermediate interactions. Solid lines indicate direct relationships (two molecules make physical contact) and dotted lines indicate indirect relationships (does not require physical contact). The scores for A), B), and C are 29, 24, and 16 respectively.
(0.48 MB PDF)

Figure S5 Plasma levels of 25 proteins stratified by population and surgical status. Dotted lines connect surgical and pre-surgical protein levels measured within the same women under both surgical and non-surgical conditions.
(0.28 MB PDF)

Table S1 Summary of all proteins identified in mouse plasma.
(0.46 MB XLS)

Table S2 Proteins increased at least 1.5-fold in plasma taken from K-ras/Pten mice compared to controls, appended with ovarian cancer cell-derived proteins assayed in human plasma. P-value for all mouse plasma ratios <0.05. The table summarizes fold difference for each protein. In addition, the ratios of secreted protein/total extract and surface protein/total extract in human ascites derived tumor cells/cancer cell lines [15] are shown for comparison for each upregulated protein in murine plasma. *Indicates cancer-only peptide(s) also observed. ELISA p-values and coefficients from logistic regression for proteins assayed in human plasma are summarized.
(0.06 MB XLS)

Table S3 Proteins decreased at least 1.5-fold in plasma taken from K-ras/Pten mice compared to controls. P-value for all mouse plasma ratios <0.05.
(0.02 MB XLS)

Table S4 Detailed information about the 58 proteins found up-regulated in the mouse model plasma experiment and enriched in the human cancer cell data.
(0.04 MB XLS)

Table S5 Categorization of upregulated IPAS data into cancer related biological processes. Each upregulated protein was assigned to a cancer related process according to its ranked network appearance determined by Ingenuity Pathway Analysis.
(0.04 MB XLS)

Table S6 Detailed GO processes for GeneGo networks shown in Figure S2.
(0.03 MB XLS)

Table S7 Clinical characteristics and protein level measurements for individual human samples.
(0.11 MB XLS)

Table S8 Summary statistics for the Generalized Estimating Equations (GEE) methods to adjust for surgical collection conditions.
(0.02 MB XLS)
