# Correction: Integrated Proteomic Analysis of Human Cancer Cells and Plasma from Tumor Bearing Mice for Ovarian Cancer Biomarker Discovery

**DOI:** 10.1371/annotation/b29e7b74-4f55-4a44-8db6-e9480bef4872

**Published:** 2009-12-11

**Authors:** Sharon J. Pitteri, Lellean JeBailey, Vitor M. Faça, Jason D. Thorpe, Melissa A. Silva, Reneé C. Ireton, Marc B. Horton, Hong Wang, Liese C. Pruitt, Qing Zhang, Kuang H. Cheng, Nicole Urban, Samir M. Hanash, Daniela M. Dinulescu

Lellean JeBailey should also be affiliated with: GeneGo Inc., St. Joseph, Michigan, United States of America. The Competing Interests statement should also be amended to read: LJ is an employee of GeneGo Inc.

