# Supplementary figures and images for: Integrated Proteomic Analysis of Human Cancer Cells and Plasma from Tumor Bearing Mice for Ovarian Cancer Biomarker Discovery
Source: PLoS One. 2009 Nov 19;4(11):e7916. doi: 10.1371/journal.pone.0007916 (PMC2775948; doi:10.1371/journal.pone.0007916)

Figure S1A

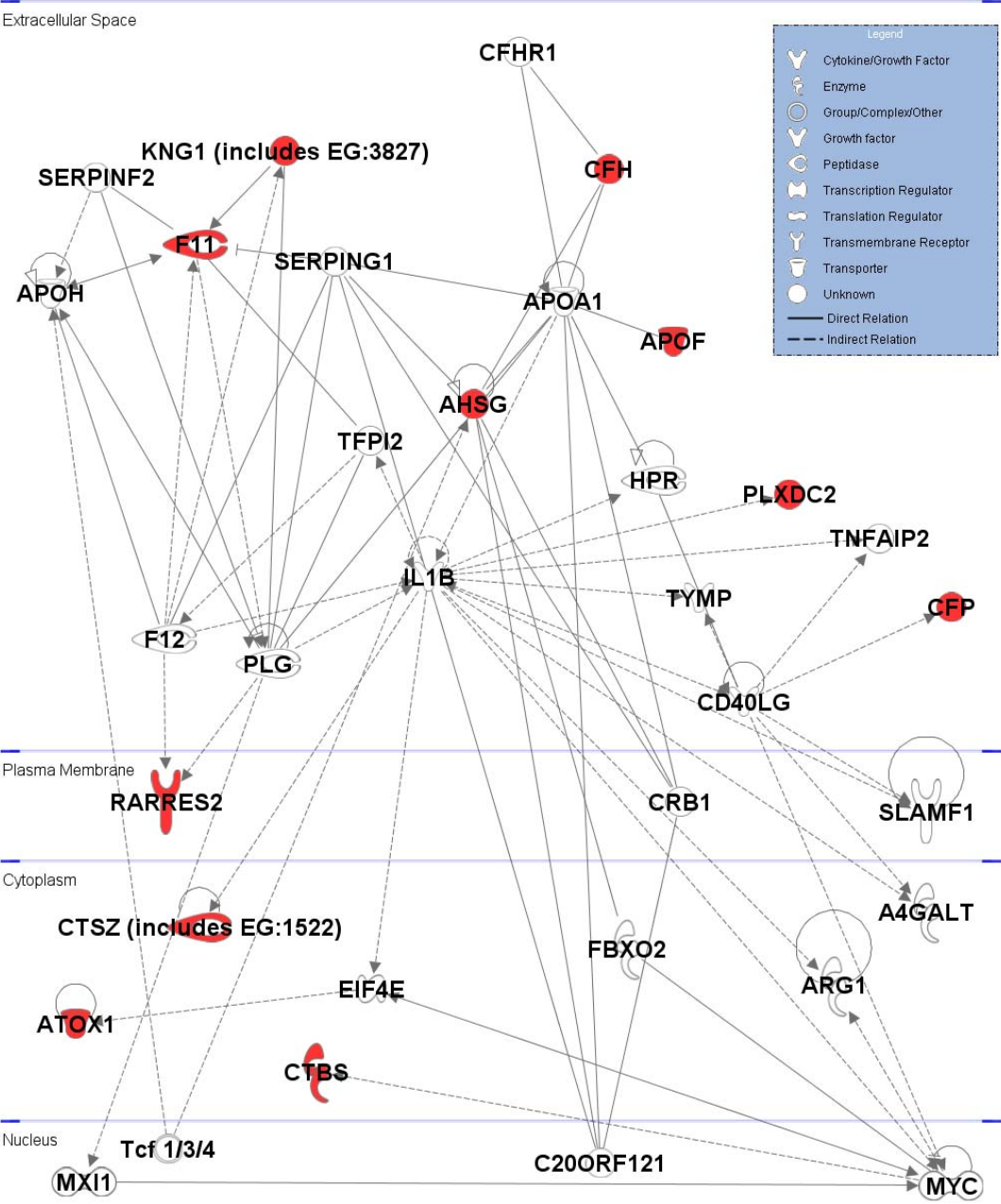

Figure S1B

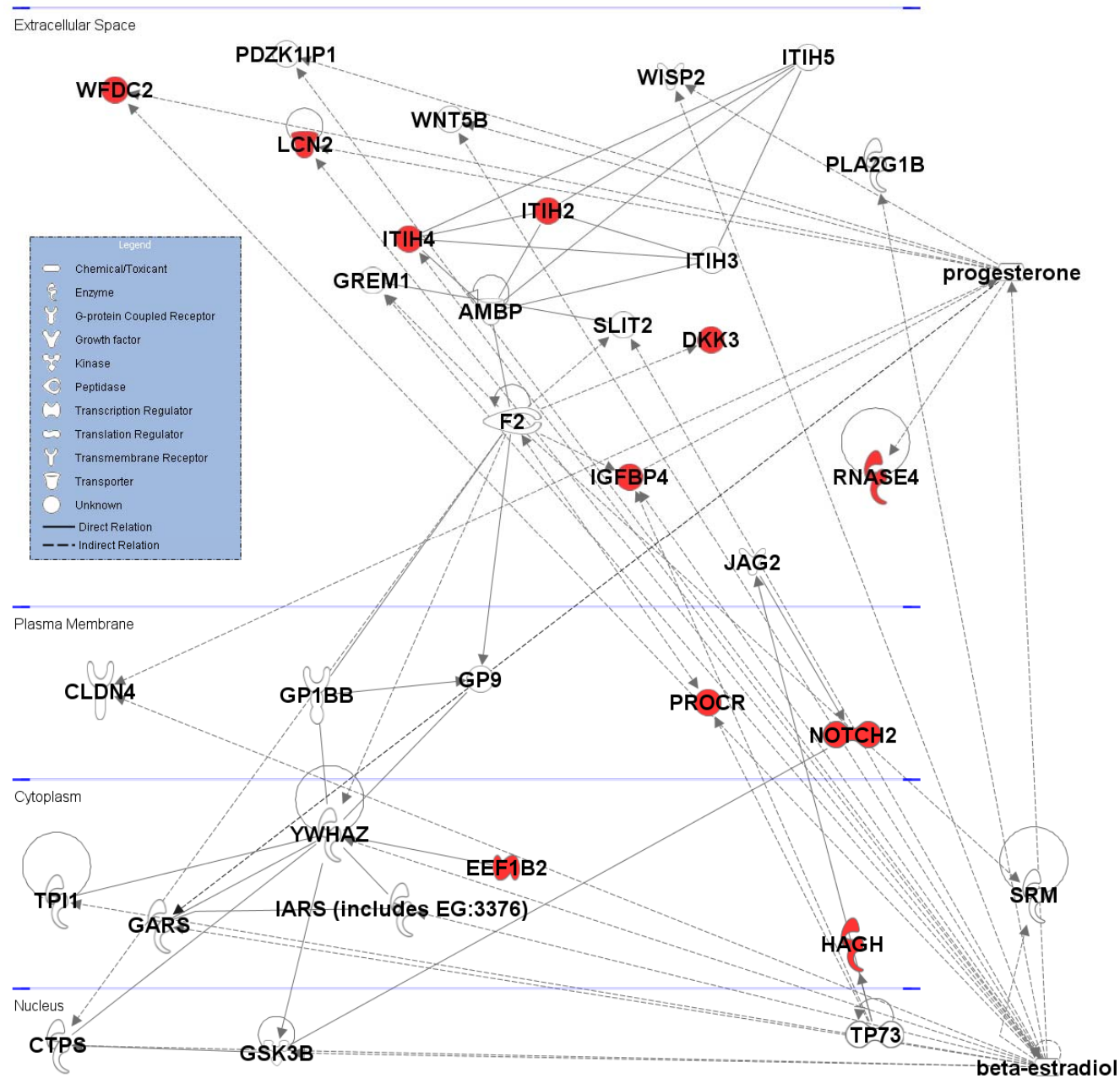

Supplement: Figure S1 — Additional networks for upregulated in mouse plasma and enriched in ovarian cancer cell data. The remaining significant networks for proteins upregulated in mouse plasma and enriched in cancer cell data assigned by Ingenuity Pathway Analysis are shown. Proteins colored in red represent proteins from the IPAS list. Non-colored proteins are those assigned by the Ingenuity database as possible intermediate interactions. Solid lines indicate direct relationships (two molecules make physical contact) and dotted lines indicate indirect relationships (does not require physical contact). The scores for A) and B) are 51 and 24 respectively. (0.38 MB PDF) [file pone.0007916.s001.pdf]

Figure S2A

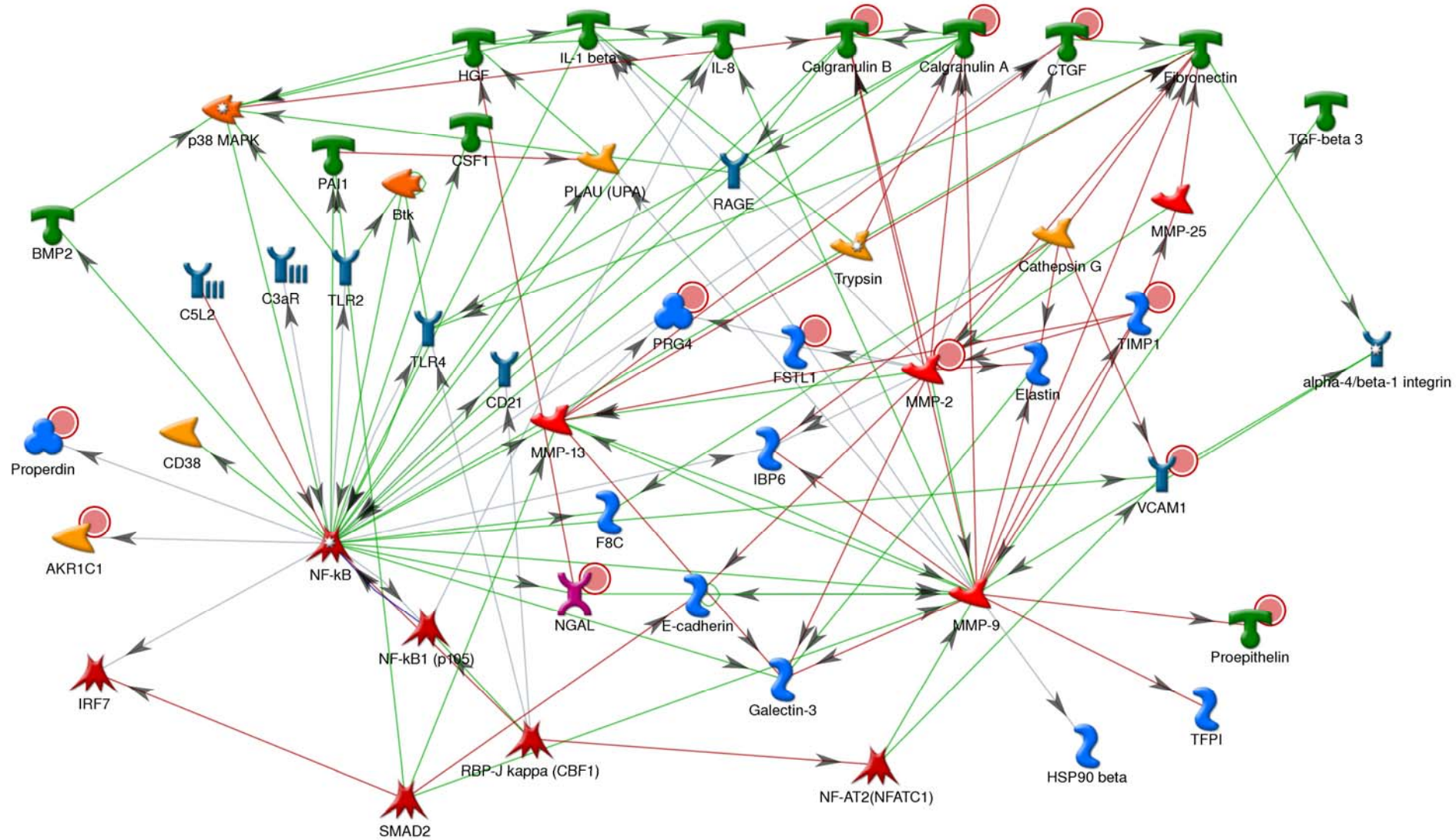

Figure S2B

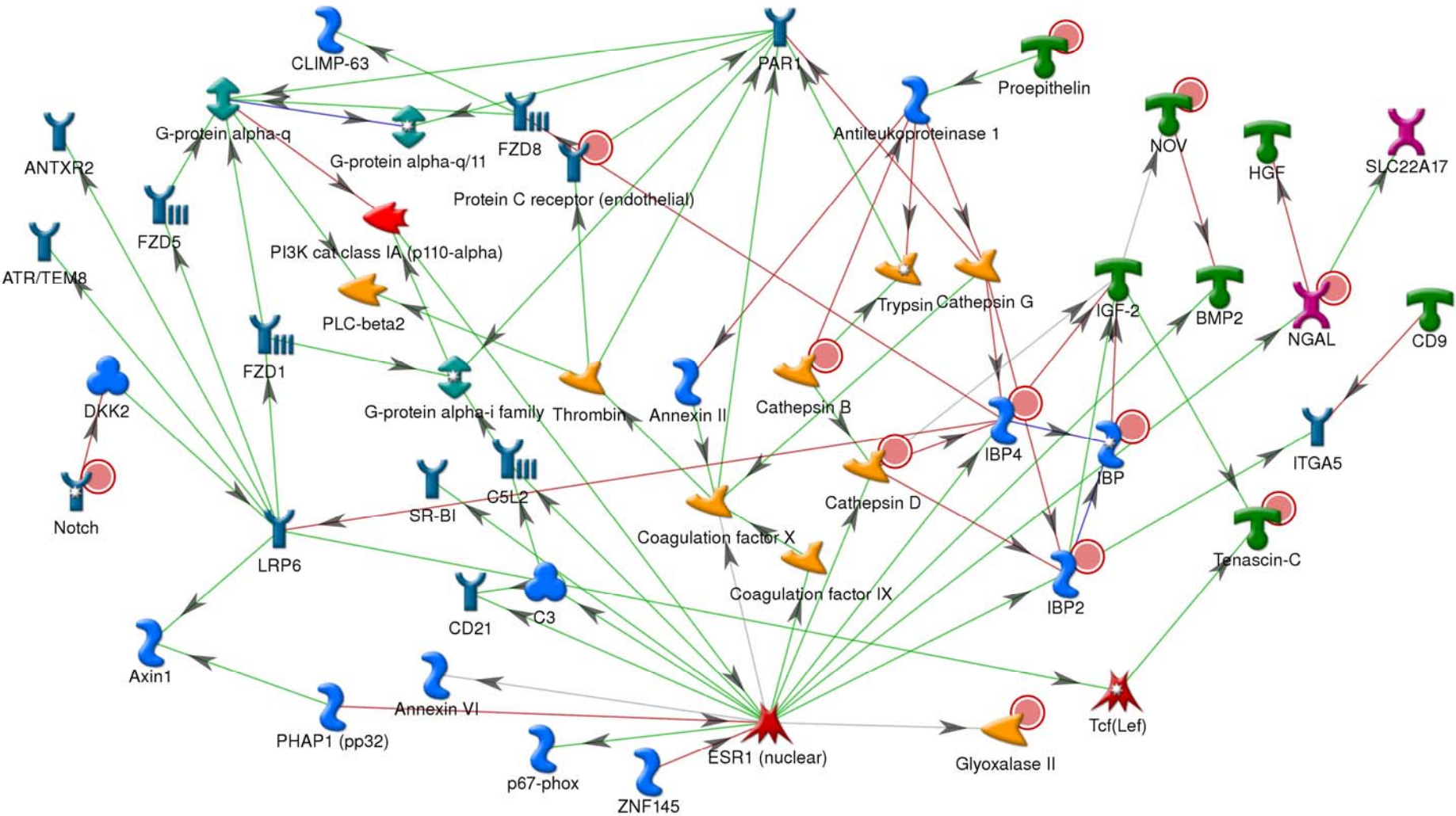

Figure S2C

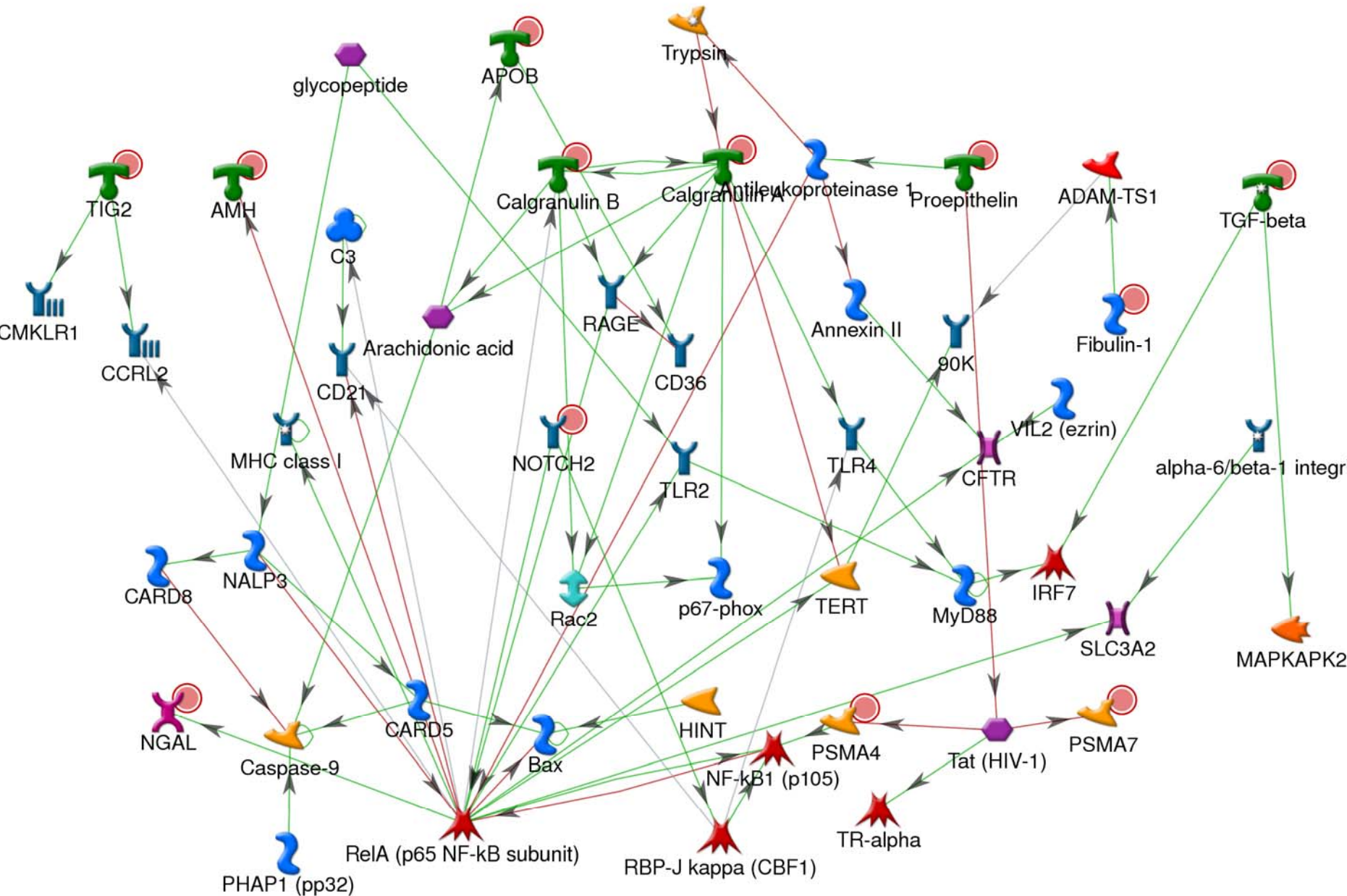

Figure S2D

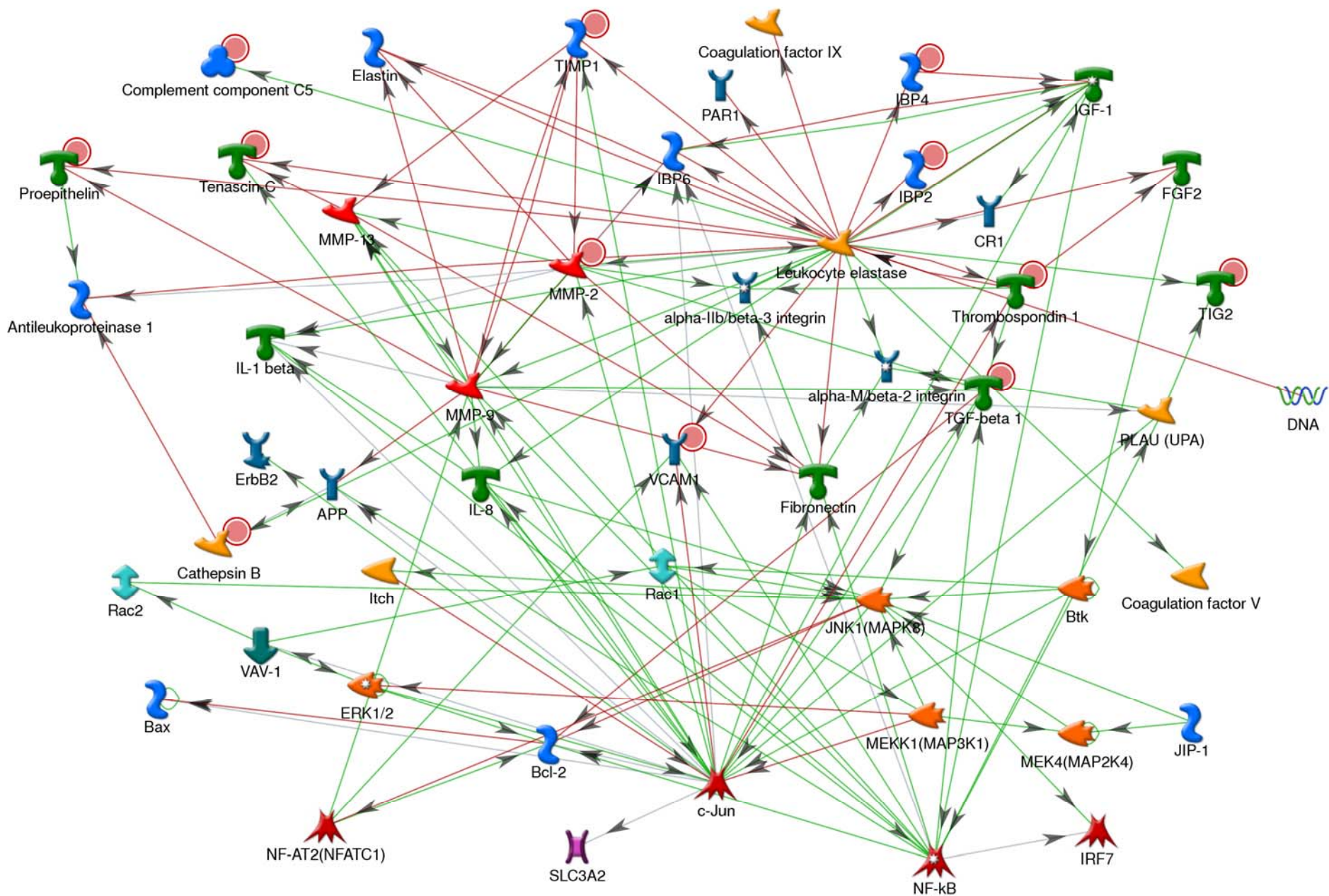

Figure S2E

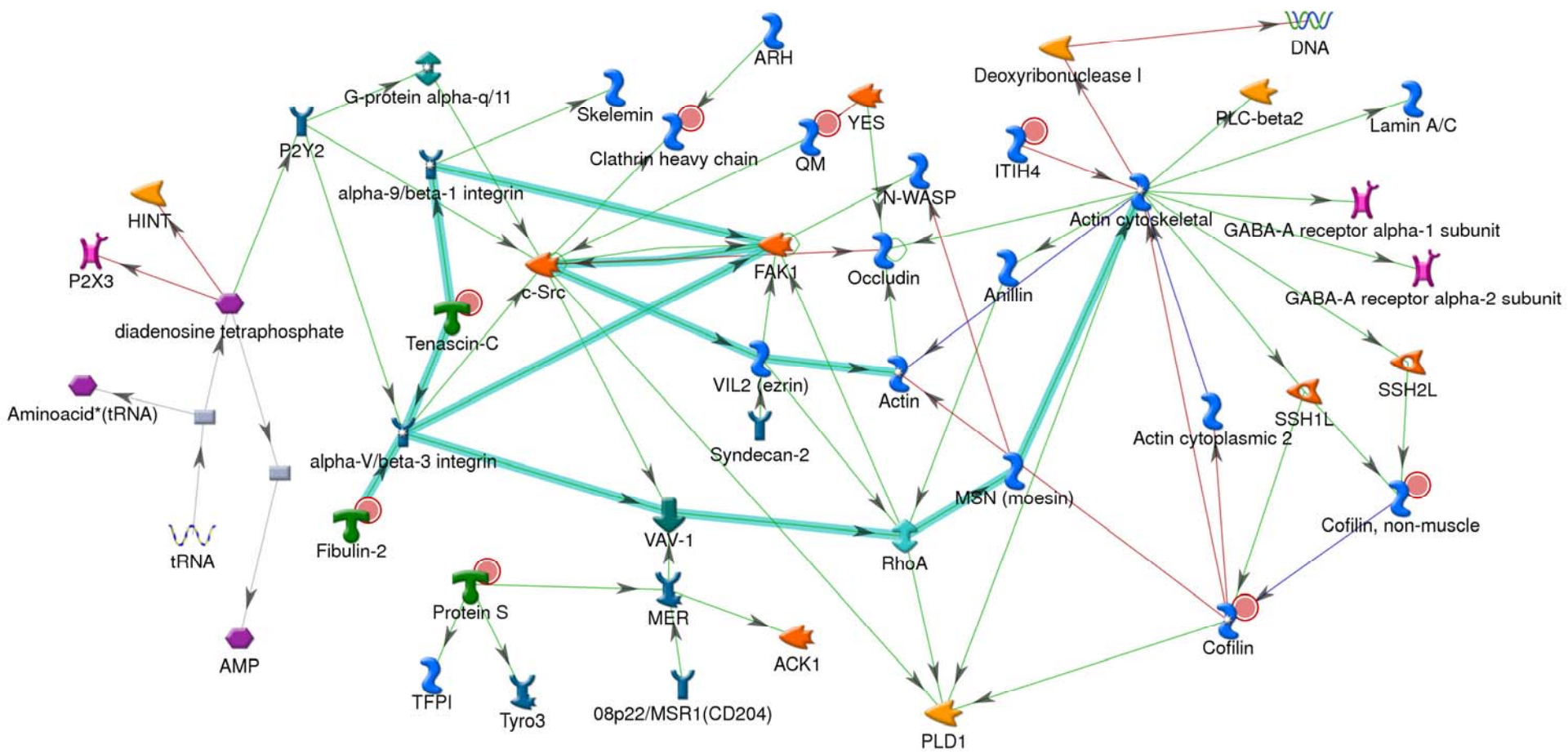

Supplement: Figure S2 — Significant networks for upregulated in mouse plasma and enriched in ovarian cancer cell data (by Metacore analysis). Five significant networks for proteins upregulated in mouse plasma and enriched in cancer cell data assigned by Metacore analysis are shown. Proteins with a pink dot represent proteins from the IPAS list. The p-values for each network are: A) 5.73e-24, B) 5.73e-24, C) 5.73e-24, D) 5.73e-24, E) 5.02e-15. (0.75 MB PDF) [file pone.0007916.s002.pdf]

Figure S3A

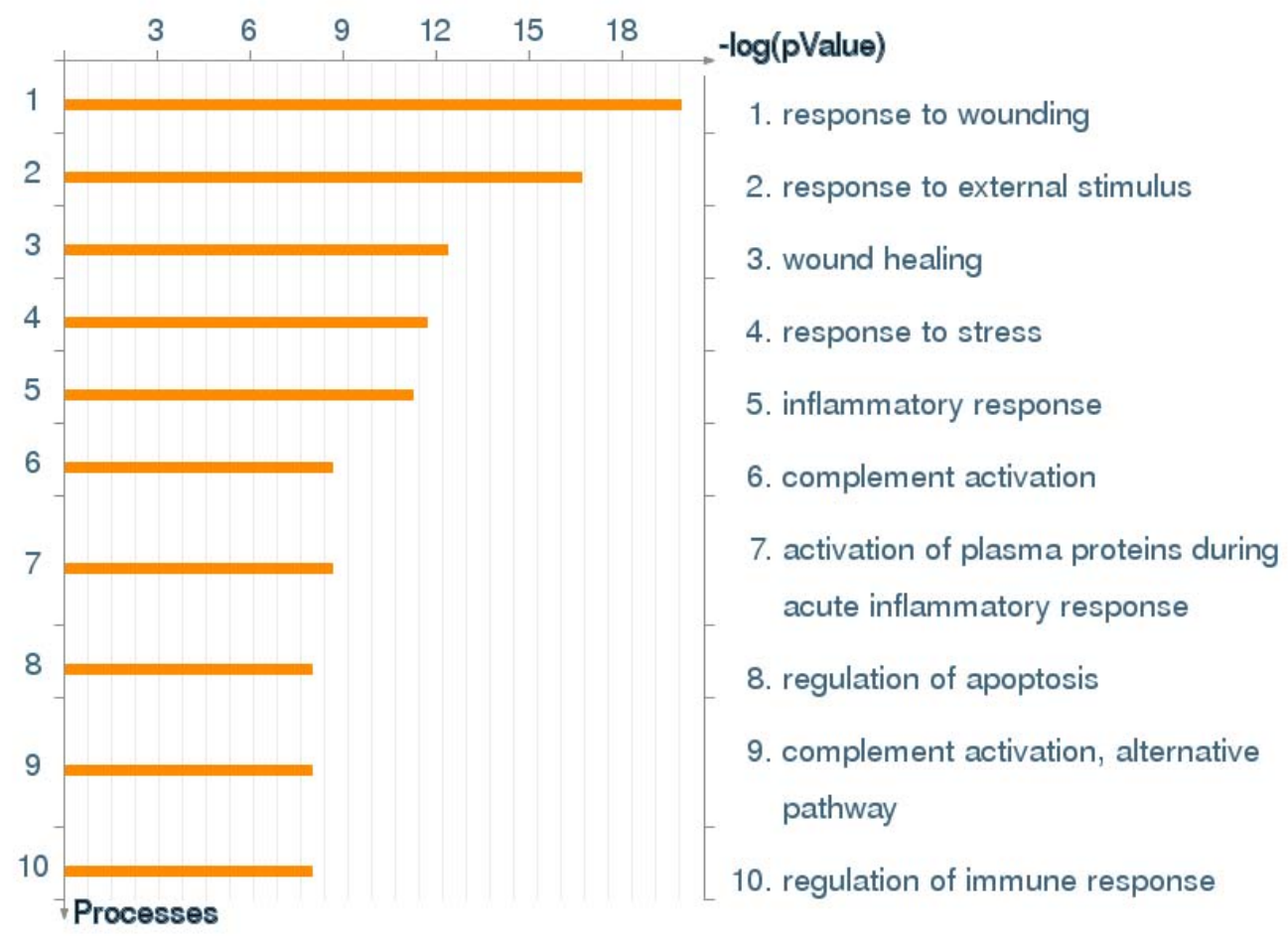

Figure S3B

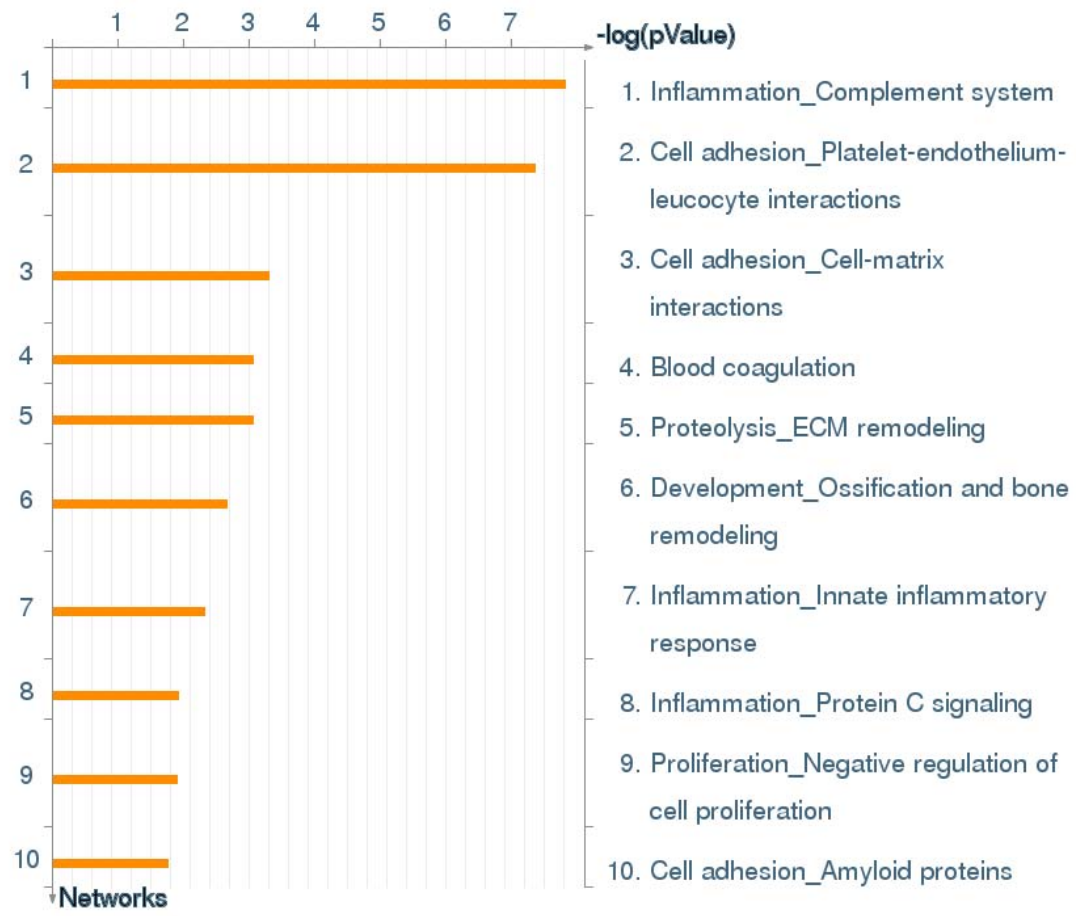

Supplement: Figure S3 — A) Gene Ontology and B) GeneGO processes for the 58 proteins upregulated in mouse plasma and enriched in ovarian cancer cell data. (0.13 MB PDF) [file pone.0007916.s003.pdf]
