# Supplementary material for: Integrated Proteomic Analysis of Human Cancer Cells and Plasma from Tumor Bearing Mice for Ovarian Cancer Biomarker Discovery
Source: PLoS One. 2009 Nov 19;4(11):e7916. doi: 10.1371/journal.pone.0007916 (PMC2775948; doi:10.1371/journal.pone.0007916)
Supplement: Figure S4 — Significant networks for proteins upregulated in mouse plasma and not found enriched in ovarian cancer cell data. The significant networks for proteins upregulated in mouse plasma, but not enriched in cancer cell data assigned by Inenuity Pathway Analysis are shown. Proteins colored in red represent proteins from the IPAS list. Non-colored proteins are those assigned by the Ingenuity database as possible intermediate interactions. Solid lines indicate direct relationships (two molecules make physical contact) and dotted lines indicate indirect relationships (does not require physical contact). The scores for A), B), and C are 29, 24, and 16 respectively. (0.48 MB PDF) [file pone.0007916.s004.pdf]

Figure S4A

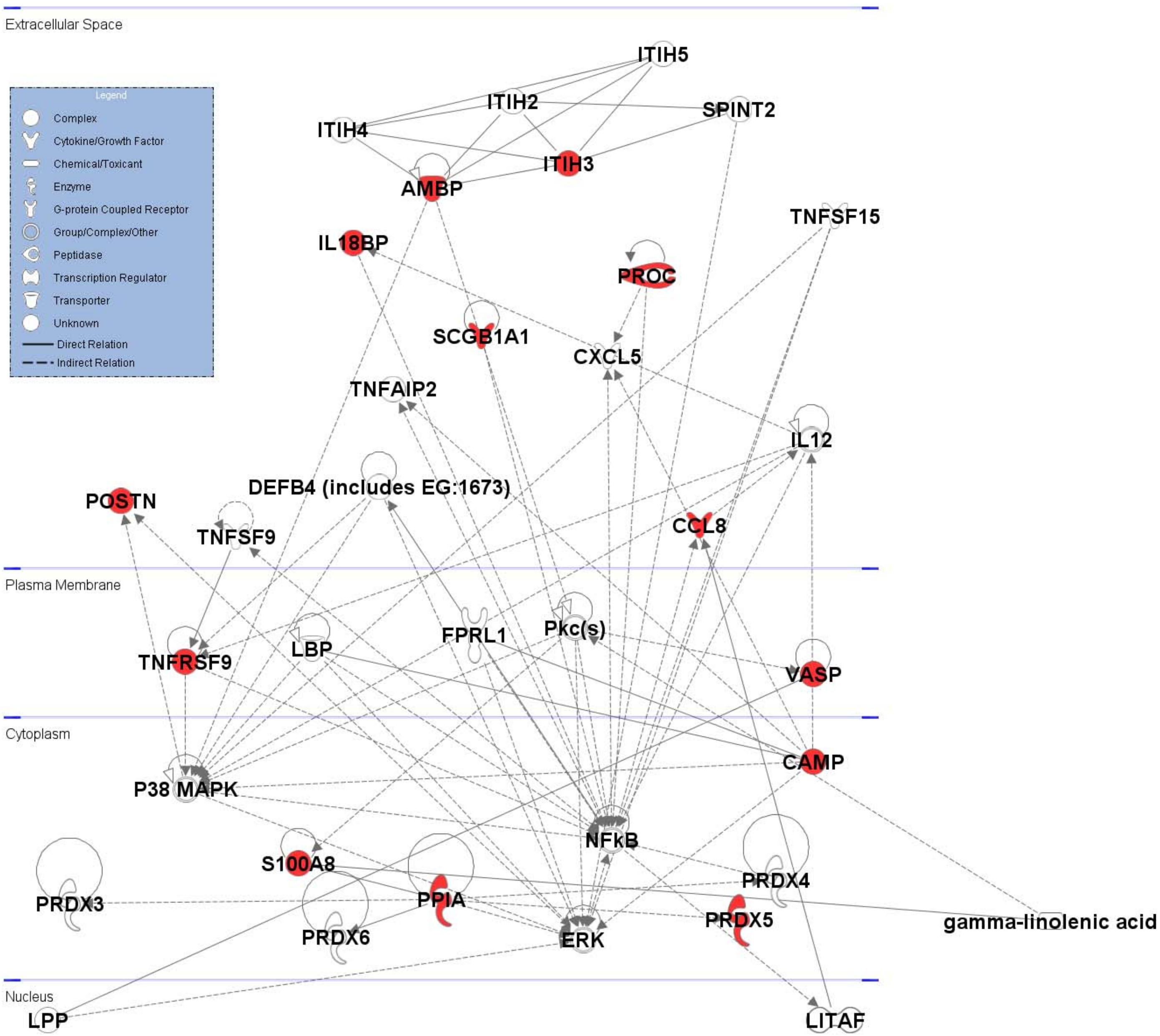

Extracellular Space
Legend
Complex
Cytokine/Growth Factor
Chemical/Toxicant
Enzyme
G-protein Coupled Receptor
Group/Complex/Other
Peptidase
Transcription Regulator
Transporter
Unknown
Direct Relation
Indirect Relation
ITIH5
ITIH2
SPINT2
ITIH4
ITIH3
AMBP
TNFSF15
IL18BP
PROC
SCGB1A1
CXCL5
TNFAIP2
IL12
DEFB4 (includes EG:1673)
POSTN
TNFSF9
CCL8
Plasma Membrane
FPRL1
Pkc(s)
LBP
TNFRSF9
VASP
Cytoplasm
CAMP
P38 MAPK
NFkB
S100A8
PRDX4
PRDX3
PPIA
gamma-linolenic acid
PRDX5
PRDX6
ERK
Nucleus
LPP
LITAF

Figure S4B

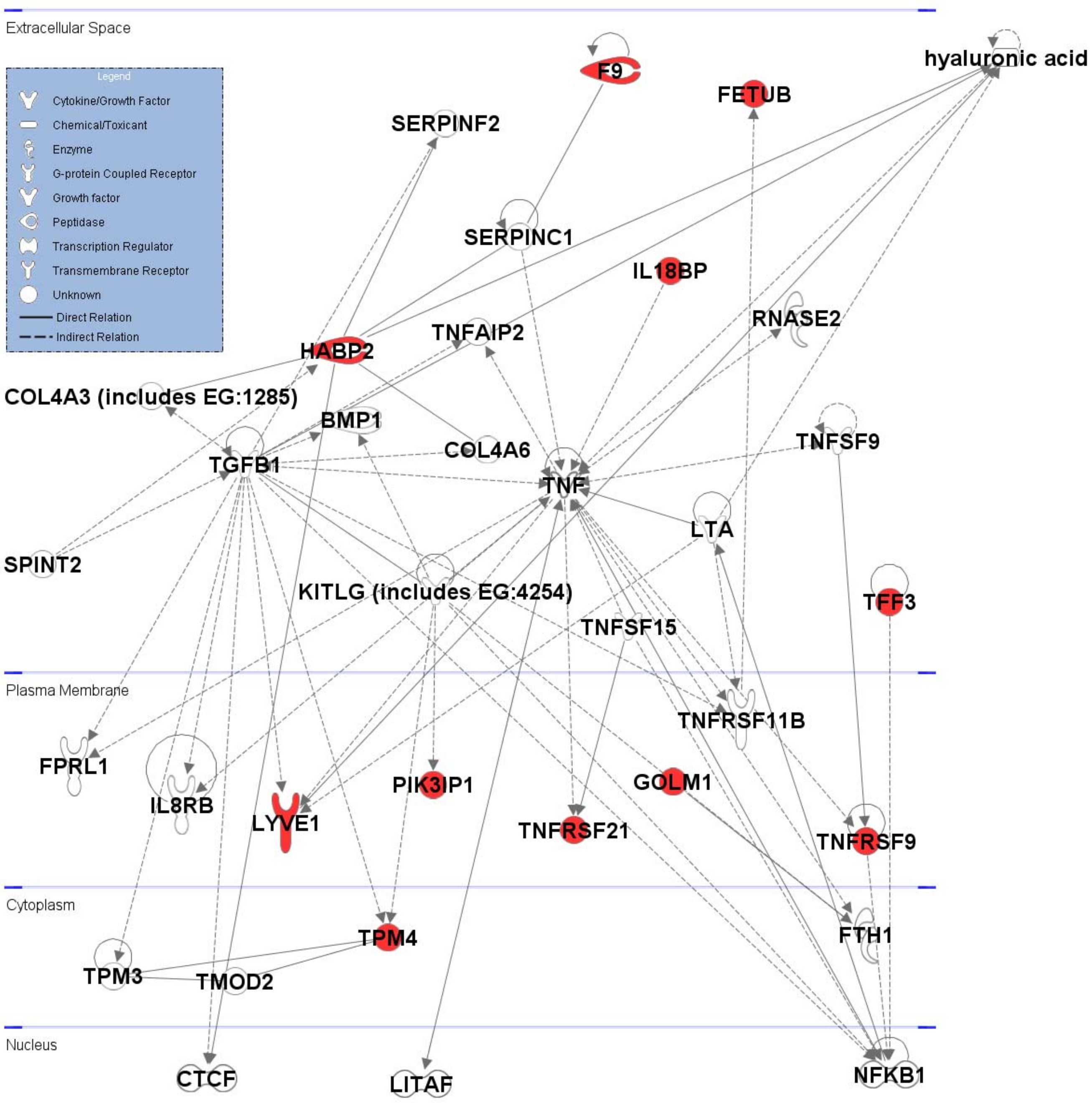
Extracellular Space
Legend
Cytokine/Growth Factor
Chemical/Toxicant
Enzyme
G-protein Coupled Receptor
Growth factor
Peptidase
Transcription Regulator
Transmembrane Receptor
Unknown
Direct Relation
Indirect Relation
F9
hyaluronic acid
FETUB
SERPINF2
SERPINC1
IL18BP
RNASE2
TNFAIP2
HABP2
COL4A3 (includes EG:1285)
BMP1
COL4A6
TNFSF9
TGFB1
TNF
LTA
SPINT2
KITLG (includes EG:4254)
TFF3
TNFSF15
Plasma Membrane
TNFRSF11B
FPRL1
IL8RB
LYVE1
PIK3IP1
GOLM1
TNFRSF21
TNFRSF9
Cytoplasm
FTH1
TPM4
TPM3
TMOD2
Nucleus
CTCF
LITAF
NFKB1

Figure S4C

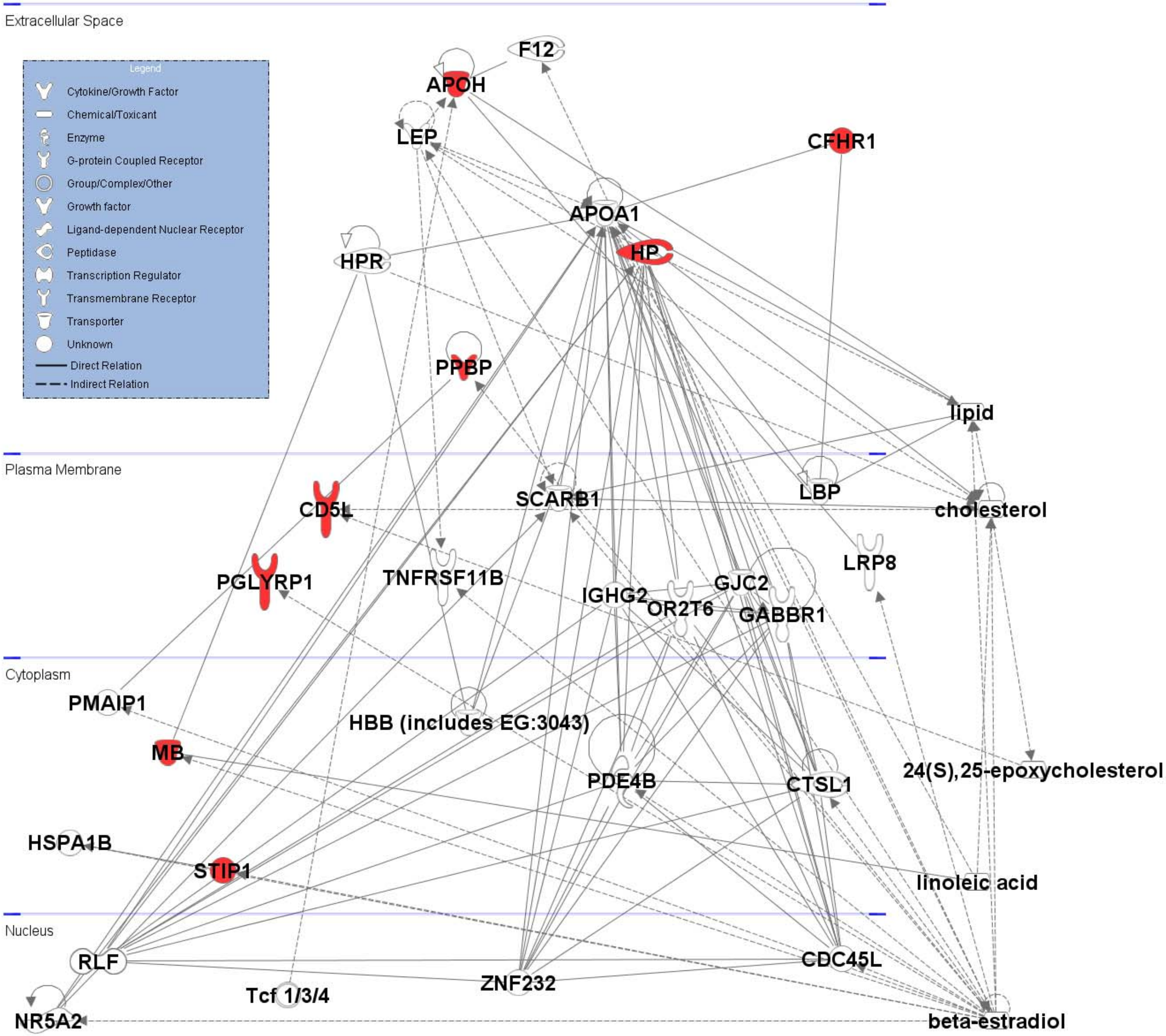

Extracellular Space
Legend
Cytokine/Growth Factor
Chemical/Toxicant
Enzyme
G-protein Coupled Receptor
Group/Complex/Other
Growth factor
Ligand-dependent Nuclear Receptor
Peptidase
Transcription Regulator
Transmembrane Receptor
Transporter
Unknown
Direct Relation
Indirect Relation
F12
APOH
LEP
CFHR1
APOA1
HPR
HP
PPBP
lipid
Plasma Membrane
CD5L
SCARB1
LBP
cholesterol
PGLYRP1
TNFRSF11B
IGHG2
OR2T6
GJC2
GABBR1
LRP8
Cytoplasm
PMAIP1
HBB (includes EG:3043)
MB
PDE4B
CTSL1
24(S),25-epoxycholesterol
HSPA1B
STIP1
linoleic acid
Nucleus
RLF
CDC45L
ZNF232
Tcf 1/3/4
NR5A2
beta-estradiol
