# Supplementary material for: Integrated Proteomic Analysis of Human Cancer Cells and Plasma from Tumor Bearing Mice for Ovarian Cancer Biomarker Discovery
Source: PLoS One. 2009 Nov 19;4(11):e7916. doi: 10.1371/journal.pone.0007916 (PMC2775948; doi:10.1371/journal.pone.0007916)
Supplement: Figure S5 — Plasma levels of 25 proteins stratified by population and surgical status. Dotted lines connect surgical and pre-surgical protein levels measured within the same women under both surgical and non-surgical conditions. (0.28 MB PDF) [file pone.0007916.s005.pdf]

Figure S5

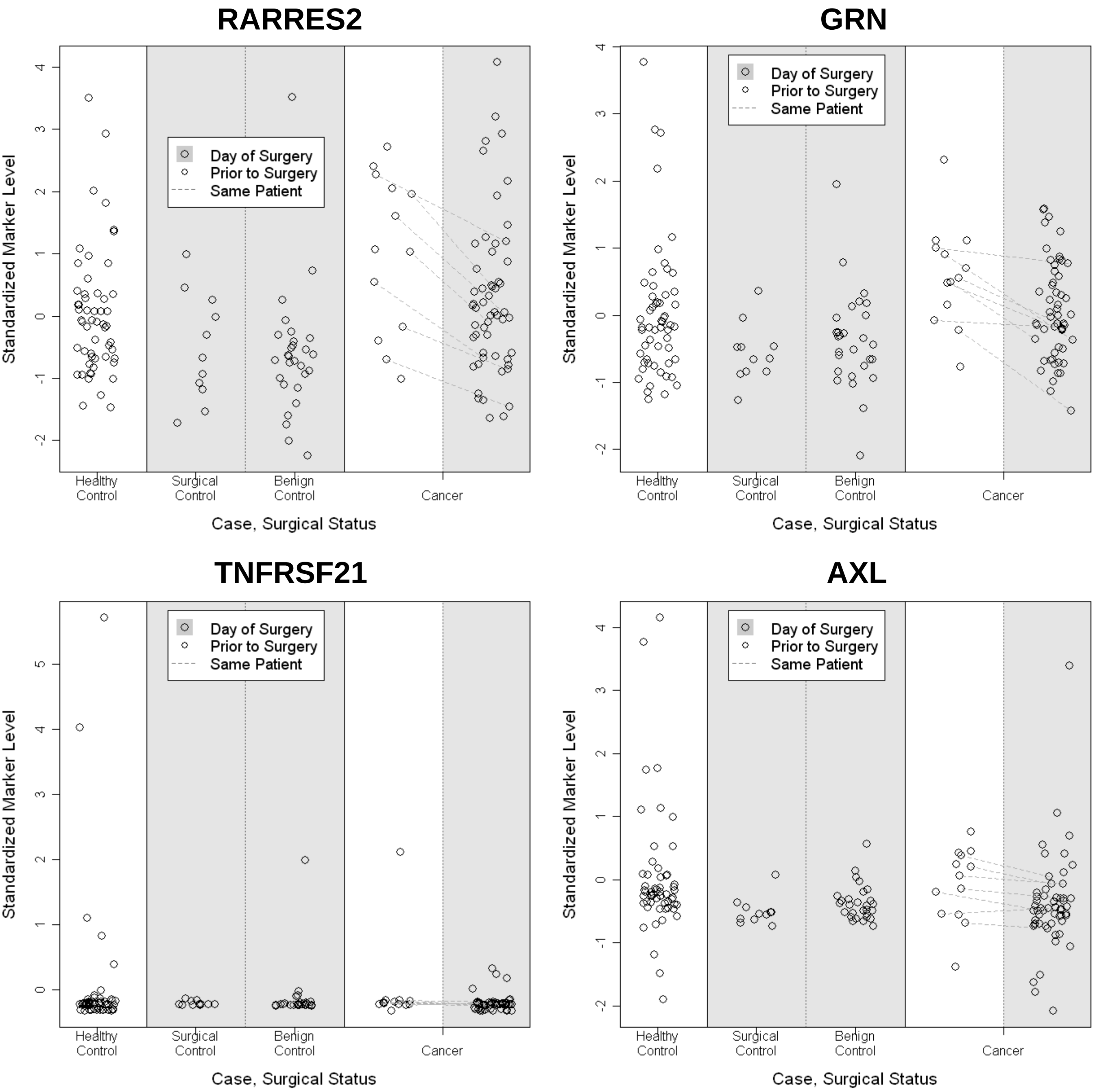
RARRES2
GRN
TNFRSF21
AXL
Day of Surgery
Prior to Surgery
Same Patient
Standardized Marker Level
Healthy Control
Surgical Control
Benign Control
Cancer
Case, Surgical Status

Figure S5

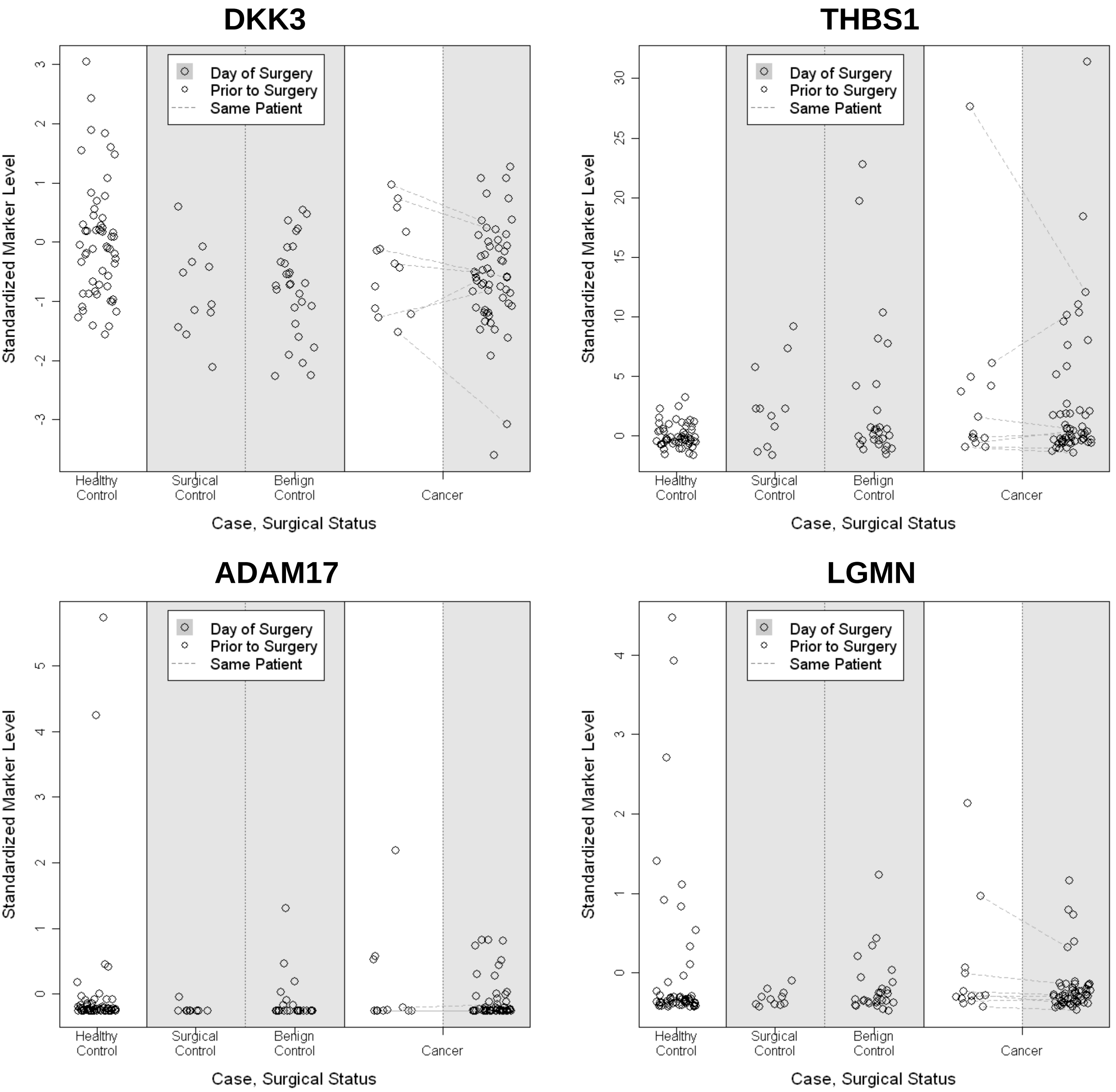
DKK3
THBS1
ADAM17
LGMN
Day of Surgery
Prior to Surgery
Same Patient
Standardized Marker Level
Healthy Control
Surgical Control
Benign Control
Cancer
Case, Surgical Status

Figure S5

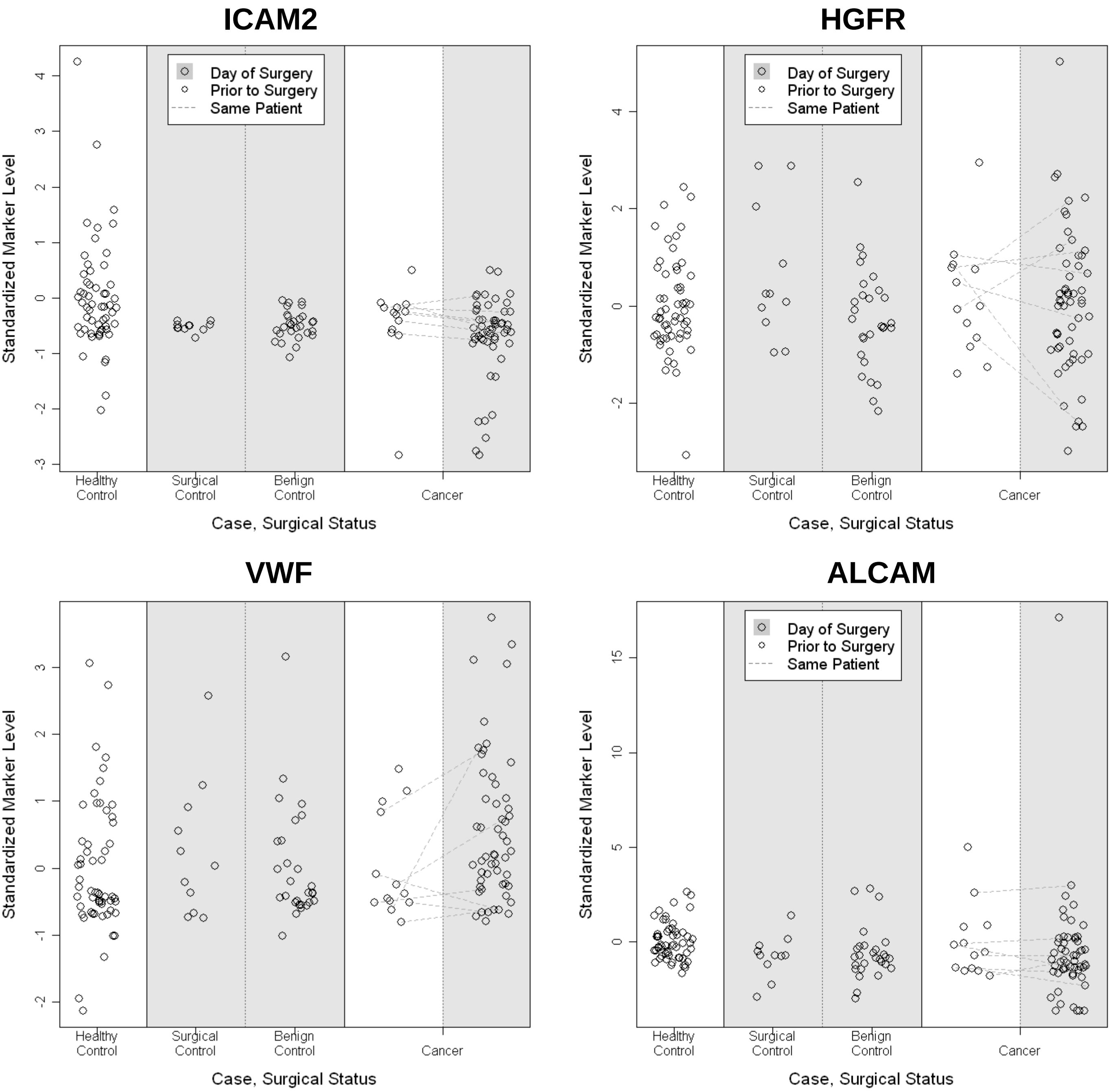
ICAM2
HGFR
VWF
ALCAM
Day of Surgery
Prior to Surgery
Same Patient
Standardized Marker Level
Healthy Control
Surgical Control
Benign Control
Cancer
Case, Surgical Status

Figure S5

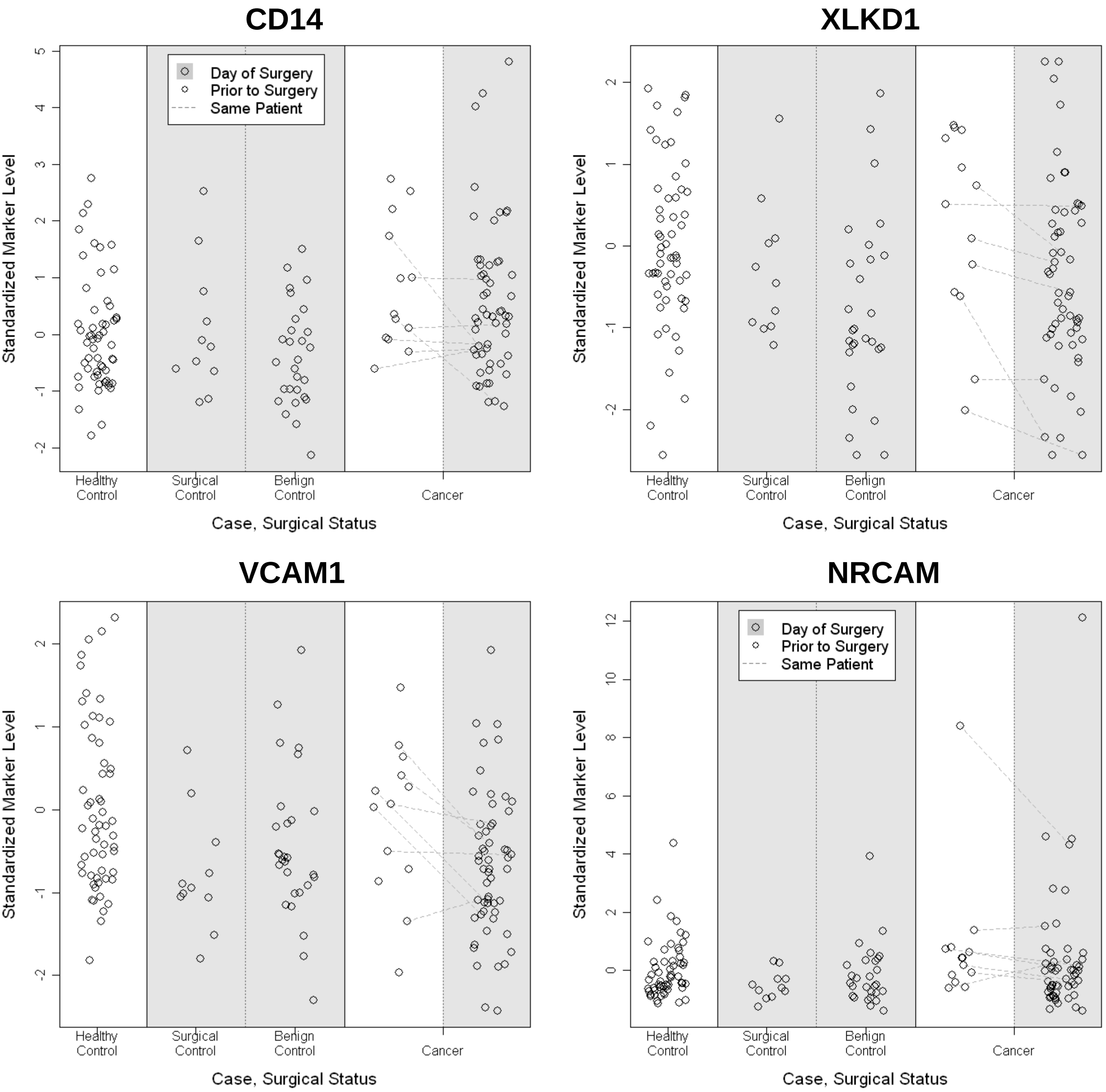

CD14
Day of Surgery
Prior to Surgery
Same Patient
Standardized Marker Level
Healthy Control
Surgical Control
Benign Control
Cancer
Case, Surgical Status
XLKD1
Standardized Marker Level
Healthy Control
Surgical Control
Benign Control
Cancer
Case, Surgical Status
VCAM1
Standardized Marker Level
Healthy Control
Surgical Control
Benign Control
Cancer
Case, Surgical Status
NRCAM
Day of Surgery
Prior to Surgery
Same Patient
Standardized Marker Level
Healthy Control
Surgical Control
Benign Control
Cancer
Case, Surgical Status

Figure S5

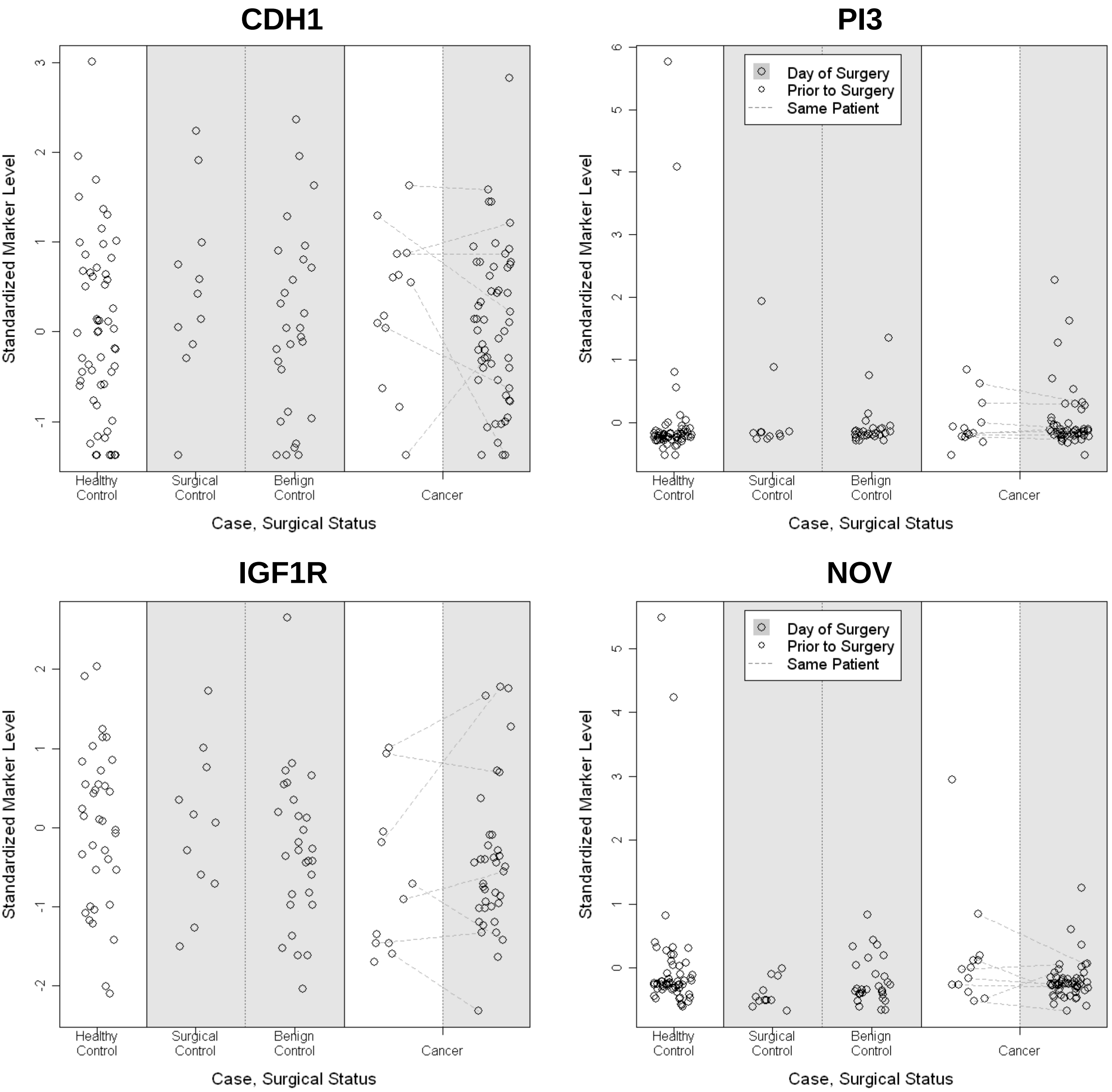

CDH1
PI3
IGF1R
NOV
Day of Surgery
Prior to Surgery
Same Patient
Standardized Marker Level
Healthy Control
Surgical Control
Benign Control
Cancer
Case, Surgical Status

Figure S5

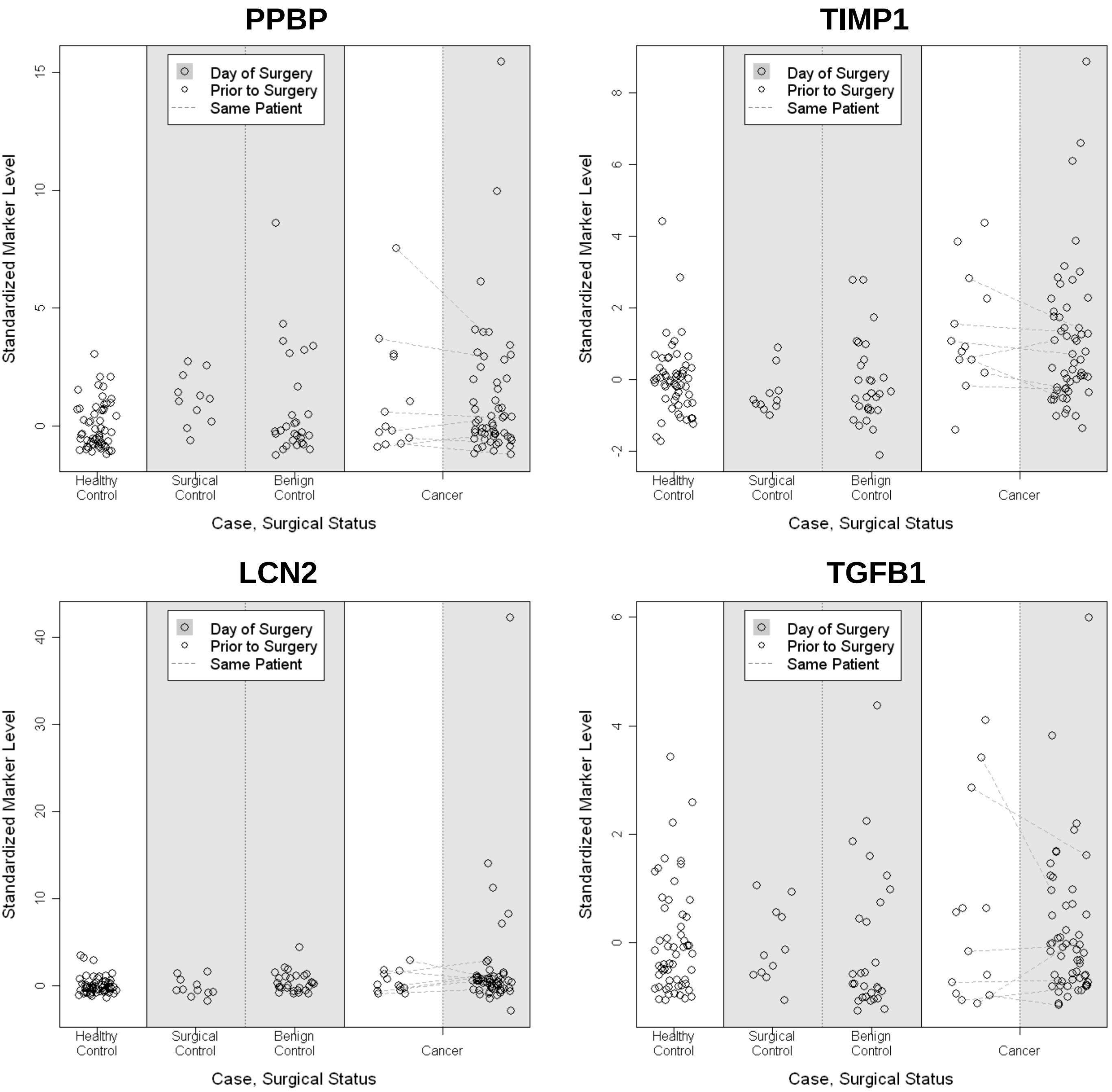

PPBP
TIMP1
LCN2
TGFB1
Day of Surgery
Prior to Surgery
Same Patient
Standardized Marker Level
Healthy Control
Surgical Control
Benign Control
Cancer
Case, Surgical Status

Figure S5

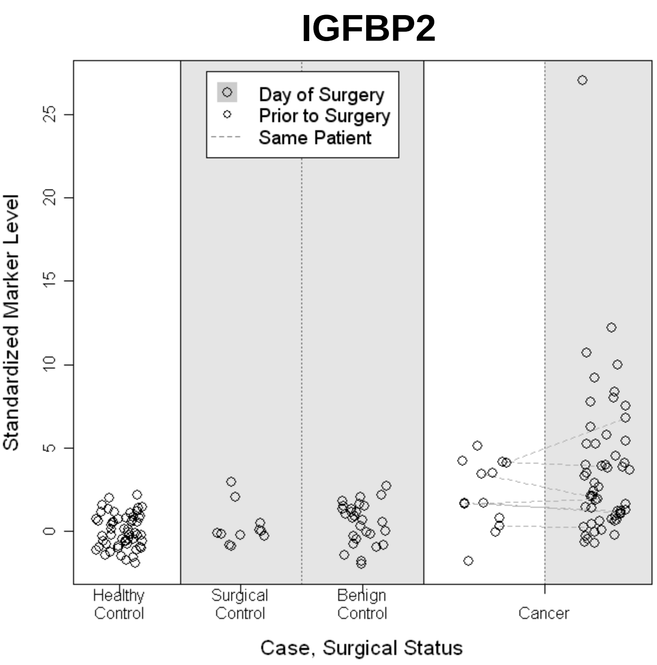
IGFBP2
Day of Surgery
Prior to Surgery
Same Patient
Standardized Marker Level
0
5
10
15
20
25
Healthy Control
Surgical Control
Benign Control
Cancer
Case, Surgical Status
